# Conductive Hydrogels for Exogenous Sensing and Cell Fate Control

**DOI:** 10.1002/adma.72866

**Published:** 2026-03-26

**Authors:** Teuku Fawzul Akbar, Carlos Alejandro Jimenez-Rodriguez, Railia Biktimirova, Ilka Hermes, Thomas Kurth, My Duyen Pham, Mikhail Tsurkan, Jens Friedrichs, Francis L.C. Morgan, Hans Kleemann, Olga Guskova, Uwe Freudenberg, Peter Fratzl, Carsten Werner, Christoph Tondera, Ivan R. Minev

**Affiliations:** 1 Division Polymer Biomaterials Science, Leibniz Institute of Polymer Research Dresden, Dresden, Germany; 2 Division Physical Chemistry and Physics of Polymers, Leibniz Institute of Polymer Research Dresden, Dresden, Germany; 3 Core Facility Electron Microscopy and Histology, Center for Molecular and Cellular Bioengineering, TUD Dresden University of Technology, Dresden, Germany; 4 Dresden Integrated Center For Applied Physics and Photonic Materials, TUD Dresden University of Technology, Dresden, Germany; 5 Division Theory of Polymers, Leibniz Institute of Polymer Research Dresden, Dresden, Germany; 6 Department of Biomaterials, Max Planck Institute of Colloids and Interfaces, Potsdam, Germany; 7 Center for Regenerative Therapies TU Dresden, TUD Dresden University of Technology, Dresden, Germany; 8 Else Kröner Fresenius Center for Digital Health, Medical Faculty Carl Gustav Carus, TUD Dresden University of Technology, Dresden, Germany

**Keywords:** bioelectronics, conductive hydrogels, electronic extracellular matrix

## Abstract

Next generation technologies linking living systems to computers will require materials built on biology, an approach that may address persistent challenges in stable and multimodal information exchange. Here, we present a semi-synthetic hydrogel, designed to emulate key features of native extracellular matrix (ECM) while offering electrically tunable functionality. We engineer interactions between sulfated glycosaminoglycans (sGAGs) and a semiconducting organic polymer (poly(3,4-ethylenedioxythiophene), PEDOT) within a soft hydrogel network (PEDOT:sGAGh). We demonstrate control over the material’s nanoarchitecture, electrochemical behavior, and biomolecular interactions. In particular, PEDOT:sGAGh exhibits affinity for bioactive proteins, including growth factors, and allows their release or retention to be modulated by low-voltage stimulation. This enables electrical control over macromolecular cues for cell differentiation, a capability not found in natural ECM or conventional conductive hydrogels. These functions are achieved with ultra-low PEDOT content (≈1 wt.%), preserving the hydrogel’s tissue-like softness and high water content. The PEDOT:sGAGh material can be integrated as a bioactive coating on electrodes, or into 3D organic electrochemical transistors (OECTs). Our results position PEDOT:sGAGh as a versatile platform for realizing biohybrid circuits that bridge molecular signaling and solid-state electronics, thus paving the way for brain-machine interfaces that operate beyond purely electrical modes of interaction.

## 1 Introduction

Advances in flexible and stretchable electronics have significantly improved the mechanical integration of devices with soft biolog-ical tissues [1–6]. These technologies enable conformal matching to complex anatomical surfaces and even accommodate tissue motion and growth [7–9]. Miniaturized implantable devices based on mesh- or web-like electrode arrays can approach cellular dimensions, an approach that may circumvent the foreign body response, while offering high resolution, 3D interfaces [10, 11]. Despite these achievements, most materials used in bioelectronics are selected primarily for their electrical properties and compatibility with microfabrication, rather than their biological function [12, 13]. This design constraint limits the extent to which bioelectronic implants can truly mimic native tissue, especially in replicating its high water content, viscoelastic mechanics, and complex biochemical behavior [14–17].

Conductive hydrogels have partially addressed this gap [18–22]. For example, hydrogels based on poly(3,4-ethylenedioxythiophene):poly(styrene sulfonate) (PEDOT:PSS) combine tissue-like mechanical properties with mixed ionic-electronic conductivity, making them attractive for conventional electrodes for pacing and sensing electroactive cells, and for the delivery of small molecule drugs [23–26]. Bioactive and conductive hydrogels incorporating polysaccharides, glycosaminoglycans (GAGs) or decellularized tissues have also been explored [27–29]. In these systems, the biological component typically serves to enhance the bulk conductivity (e.g., through doping an organic semiconductor component) or to improve the cytocompatibility of supports for tissue regeneration or differentiation [30, 31]. Conductive hydrogels can also be polymerized or self-assembled in situ, including within living tissue, where they coexist with native extracellular matrix (ECM) and cells [32–35].

**Figure 1:**
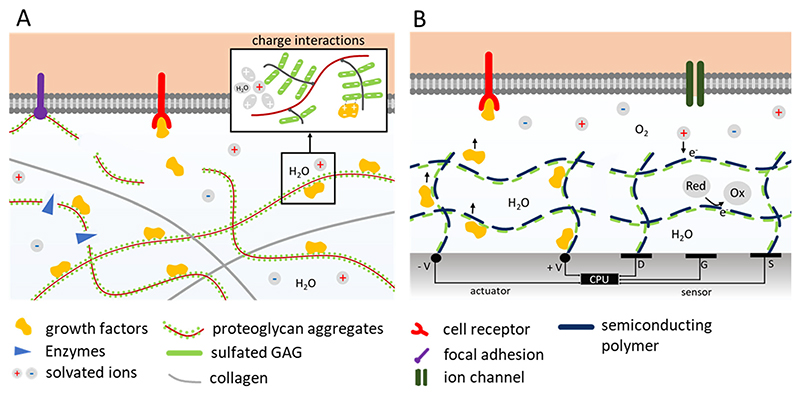
A biomimetic approach to tissue-electronics interfaces. (A) Dynamic connection between cells and ECM. Enzymes (e.g., matrix metalloproteinases and glucosidases) degrade proteoglycan aggregates and sulfated glycosaminoglycans (sGAG) trigger the release of growth factors and cytokines. sGAG such as heparin can contain both sulfate and sulfonate groups, however for simplicity we will refer to them collectively as sulfate groups in the following. The released signaling molecules bind with cell receptors activating downstream signaling pathways. (B) In a bioinspired hydrogel, a semiconducting polymer (e.g., PEDOT) is combined with selected, charge bearing components of the native ECM (e.g., sGAG). The conductive states of the resulting material can be modulated by injection of electronic charges or by ionic/molecular fluxes. This enables PEDOT:sGAGh to act as either as molecular actuator or sensor in bioelectronic interfaces.

Despite recent progress, truly tissue-mimetic bioelectronic materials remain elusive. Toward this goal, material systems must go beyond mimicking the static composition of biological tissues; they must also replicate dynamic biochemical functions. For example, in native tissues, the ECM not only provides adhesion and mechanical support but also actively regulates cell behavior by modulating the local availability of soluble signaling molecules, including growth factors and cytokines (Figure 1A). This regulation is governed by their assembly and disassembly (through semi-specific electrostatic interactions) with complex macromolecular networks composed of proteins and proteoglycans [36–38]. Seeking to emulate these functions, combining ECM components with organic semi-conductors may offer a way to control biological interactions electronically. The organic semiconductor component can modulate the material’s capacity to bind and release signaling molecules, effectively linking electronic inputs to biochemical outputs (Figure 1B). Conversely, charge complexation, redox reactions and ionic fluxes triggered by molecular interactions may be harnessed to modulate the material’s electronic properties. These electro-biochemical couplings within synthetic, decellularized or even living ECMs represent a largely unexplored frontier with significant potential for advancing bioelectronic interfaces.

Here we present an ECM-inspired metamaterial based on thoroughly defined sulfated glycosaminoglycan-containing hydrogels (sGAGh), which have previously been shown to bind and release ECM-associated growth factors and cytokines [39]. To enable dynamic, on-demand control of these interactions, we introduce dual electronic-ionic conductivity by incorporating PEDOT into the sGAGh template. The anionic charge density and amphiphilicity of the sGAGh template were found to be key parameters governing the incorporation of PEDOT and thus the nanostructure and electrochemical behavior of the resulting PEDOT:sGAGh material. Within electrochemical conditions compatible with biological systems (i.e., at potentials within the water window), we demonstrate that electrical stimulation can tune the release or retention of various growth factors, likely by modulating the electrostatic landscape within the material. We show that PEDOT:sGAGh can also function as an oxygen sensor, and when integrated into a biohybrid circuit, it can link the detection of physiological signals to electronically regulated cell fate control.

## 2 Results

### 2.1 sGAGh as Templates for PEDOT Synthesis

**Figure 2:**
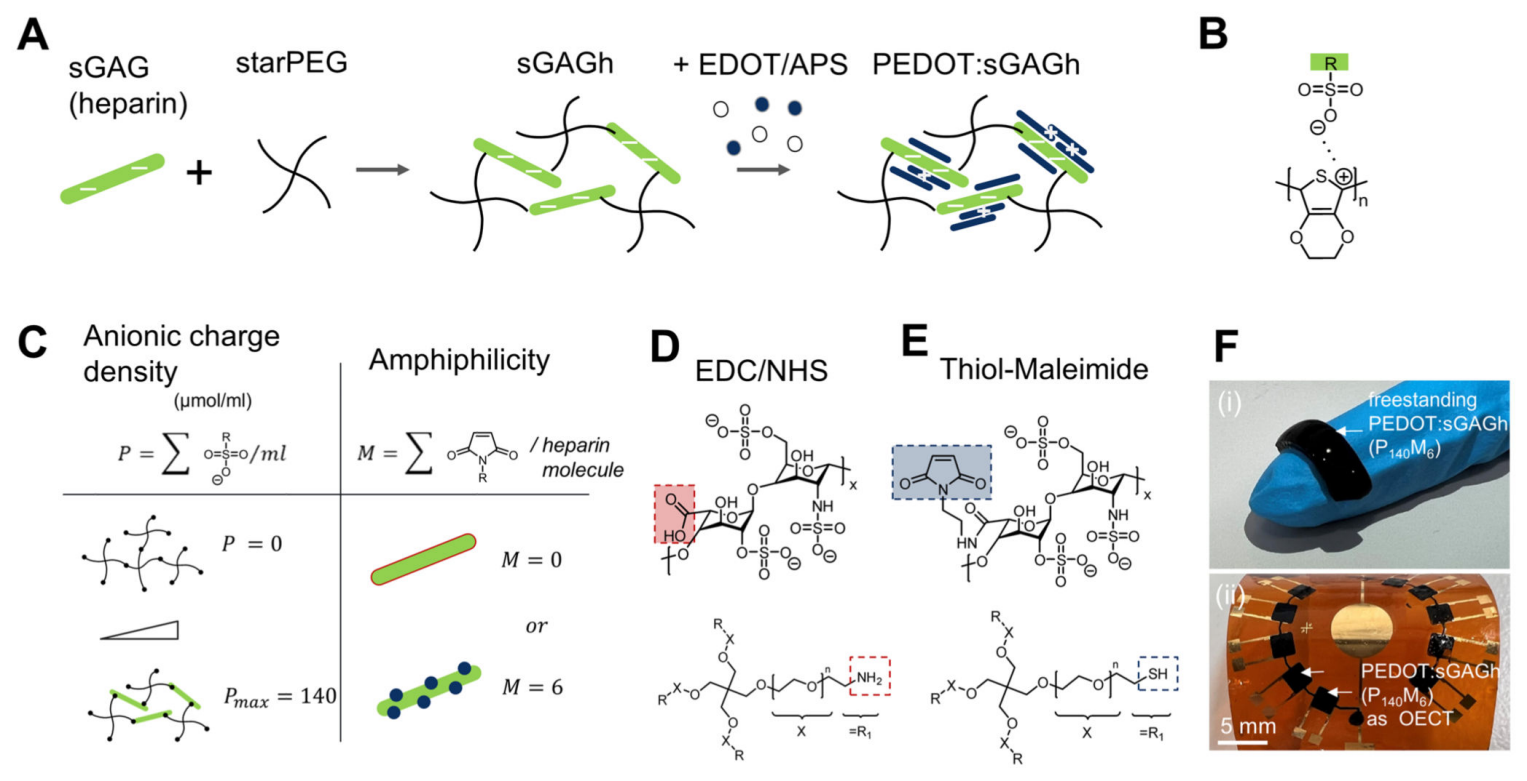
Synthesis and parametrization of sGAGh and PEDOT:sGAGh. (A) Heparin sulfate (11 500 g/mol) is cross linked with starPEG (10 000 g/mol) to create sGAGh templates. Subsequent polymerization of PEDOT proceeds by diffusion-controlled oxidative polymerization initiated by ammonium persulfate (APS) in the presence of hydrochloric acid. (B) Anionic sulfate groups act as countercharges for PEDOT. (C) The anionic charge density parameter *P* (μmol/mL) determines the amount of sulfate groups present in a volume of hydrogel. To preserve cross link density, changes in *P* (i.e., changes in heparin content) are compensated by additional starPEG functionalized with complementary (maleimide) reactive groups. The amphiphilicity parameter *M* describes the degree of maleimide functionalization of heparin. Hydrogels with *M* = 0 are produced via EDC/NHS chemistry and those where *M* = 6 are produced by linking maleimide functionalized heparin with thiol functionalized starPEG. Maintaining a constant stochiometric ratio between starPEG and heparin molecules ensures cross link density is not affected by changing *M*. However, since the two cross linking systems resulted in slightly different swelling, changing *M* results in slight deviation (< 10%) of the *P* parameter. (D) Functional groups participating in EDC/NHS chemistry. (E) Functional groups participating in thiol-maleimide chemistry. (F) Samples of PEDOT:sGAGh, (i) as a standalone material and (ii) as channels in organic electrochemical transistors (OECTs) fabricated on polyimide.

We begin by assembling hydrogels from a highly sulfated GAG (heparin) and four-armed polyethylene glycol (starPEG). The resultant hydrogels (sGAGh), serve as molecular templates for the oxidative polymerization of PEDOT, a p-type semiconductive polymer (Figure 2A; Figure S1). Electrostatic complexation between sulfate groups and PEDOT render the resulting PEDOT:sGAGh hydrogels conductive (Figure 2B). To systematically modulate the interaction of PEDOT with the sGAGh template, we control two key parameters: anionic charge density (*P*) and amphiphilicity (*M*). The *P* parameter describes the concentration of sulfate groups (0–140 μmol/mL), and is adjusted by varying the heparin content (Figure 2C; Table S1) [38]. A hydrogel with *P* = 0 μmol/mL is thus a network of starPEG without heparin. A hydrogel with *P* = 140 μmol/mL corresponds to a 1:1 molar ratio between starPEG and heparin molecules. The *M* parameter reflects the average number of maleimide substitutions on each heparin molecule replacing the more polar carboxyl groups (Figures S2 and S3). In our system *M* is set to either 0 or 6. Calculations of the hydration free energy and the Hildebrand solubility parameter indicate that maleimide substitution reduces the water affinity of heparin (Figure S4).

Two orthogonal crosslinking strategies enable control over *P* and *M* parameters. The first uses EDC/NHS coupling to link amino-functionalized starPEG with carboxyl groups on heparin (Figure 2D). The second employs a thiol–maleimide click reaction between thiol-functionalized starPEG and maleimide-modified heparin (Figure 2E). Both strategies yield covalently crosslinked sGAGh templates that remain stable and visibly turn black following PEDOT polymerization. We thus produced a range of formulations, subsequently referred to by their *M* and *P* parameters, as freestanding or substrate integrated PEDOT:sGAGh (Figure 2F).

### 2.2 PEDOT Induces Nanostructure Reorganization in sGAGh

**Figure 3:**
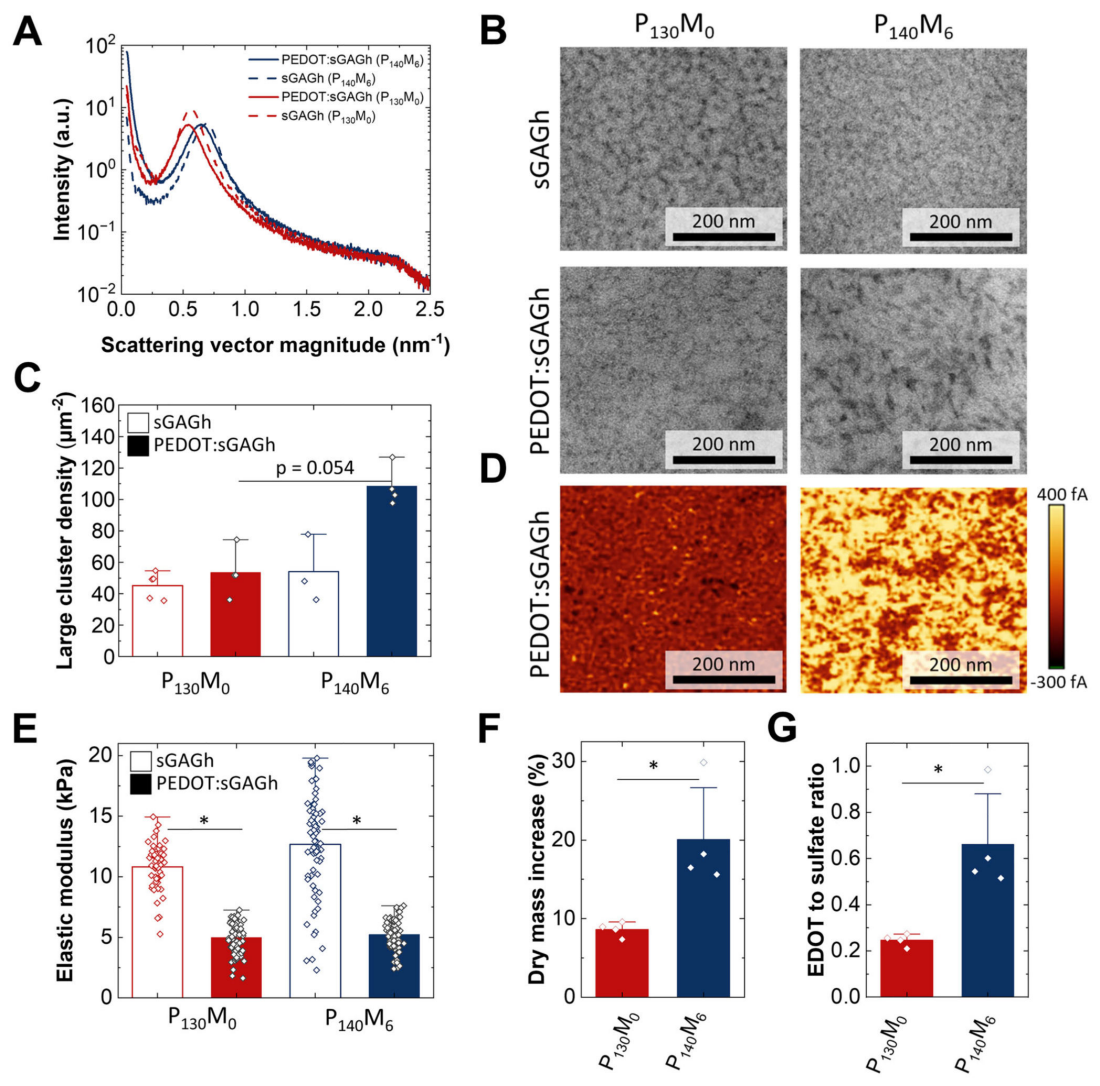
Nanostructure and composition of PEDOT:sGAGh. (A) Representative SAXS plots of dehydrated samples of sGAGh and PEDOT:sGAGh (P_130_M_0_ and P_140_M_6_). (B) Representative TEM sections of dehydrated samples of sGAGh and PEDOT:sGAGh (P_130_M_0_ and P_140_M_6_). (C) Quantitative analysis of large sGAG-rich cluster (>500 nm^2^) distribution. (D) cAFM performed at -20 mV sample voltage reveals a conductive percolating network for the P_140_M_6_ PEDOT:sGAGh. (E) Elastic moduli before and after PEDOT integration, obtained by AFM indentation with a 10 μm diameter spherical bead. (F) Dry mass increase following polymerization of PEDOT. (G) Ratio of EDOT repeating units to sulfate groups calculated under the assumption that dry mass change is only due to PEDOT incorporation. Bar plots (C, E-G) show individual data points, averages and + 1 S.D. Statistical significance was assessed using Kruskal-Wallis ANOVA, followed by a Conover’s post-hoc test for multiple comparisons. Differences were considered statistically significant at *p* < 0.05 and are indicated with *.

To understand the influence of PEDOT on the sGAGh template, a detailed investigation of nanostructure was performed. Small Angle x-ray Scattering (SAXS) in the dehydrated state revealed a semicrystalline structure for the pristine sGAGh template where amorphous interstitial spaces are bound by crystalline domains (Figure 3A). The characteristic size of the interstitial spaces is estimated in the range of 9–12 nm (Figure S5). Transmission electron microscopy (TEM) of sections stained with uranyl acetate (which binds to negatively charged sulfate groups) allowed us to visualize these structures at the nanoscale, corroborating their size (Figure 3B). Upon addition of PEDOT, the semicrystalline structure is maintained. Furthermore, PEDOT does not appear to significantly change the size of interstitial spaces estimated via SAXS (Figure S5), but results in the appearance of larger electron-dense clusters (defined as particles larger than 500 nm^2^), notably in the P_140_M_6_ hydrogels (Figure 3B,C). Conductive AFM (cAFM) reveals that these large clusters transport electronic charge indicating they contain PEDOT in addition to sGAG (Figure 3D; Figure S6). Interestingly, we observe that incorporation of PEDOT decreases the average elastic modulus of hydrogels by a factor of approximately 2 (Figure 3E) when measured via AFM-indentation. Rheological measurements however showed no significant difference after incorporation of PEDOT, likely reflecting differences between local and bulk properties of the networks. Rheological frequency and strain sweeps showed constant bulk shear moduli with the expected elastic behavior of covalently cross-linked hydrogels (Figure S7).

To understand if the amphiphilicity (*M*) plays a role in the formation of PEDOT:sGAGh, we performed gravimetric analysis. As illustrated in Figure 3F, incorporation of PEDOT leads to an increase in the dry mass, which is more pronounced for the P_140_M_6_ material. Using the gravimetric data and stoichiometry, we calculated that for every sulfate group in P_130_M_0_ hydrogels there are on average 0.25 EDOT repeating units, while in P_140_M_6_ hydrogels there are 0.65 EDOT repeat units for each sulfate group (Figure 3G). In PEDOT:sGAGh swollen in PBS, the amount of PEDOT was estimated at approximately 0.4 wt.% (P_130_M_0_) and 1.0 wt.% (P_140_M_6_).

We propose that during polymerization, EDOT enters sGAGh via the interstitial spaces and polymerizes on sGAG-rich crystalline domains, thus forming conductive clusters. The amphiphilicity parameter *M* appears to influence the overall amount of PEDOT integrated in the template. This may be due to reduced polarity and reduced water affinity of heparin caused by substitution of carboxylic with maleimide groups. Thus, maleimide groups in P_140_M_6_ may act as hydrophobic nucleation sites for enhanced PEDOT templating [40]. Following nucleation, clusters may grow by *π-π* stacking of PEDOT chains leading to non-destructive growth on the template [41–43]. When large PEDOT-rich clusters are formed at sufficiently high density (P_140_M_6_), an electrically conductive percolating network is formed throughout the bulk of the hydrogel, as suggested by cAFM. By bridging elastic domains, localized PEDOT growth may also effectively reduce the concentration of elastically active sGAGh network elements, leading to a reduction in the local apparent elastic modulus [44].

### 2.3 Anionic Charge Density and Amphiphilicity Control Bulk Electrical Properties

**Figure 4:**
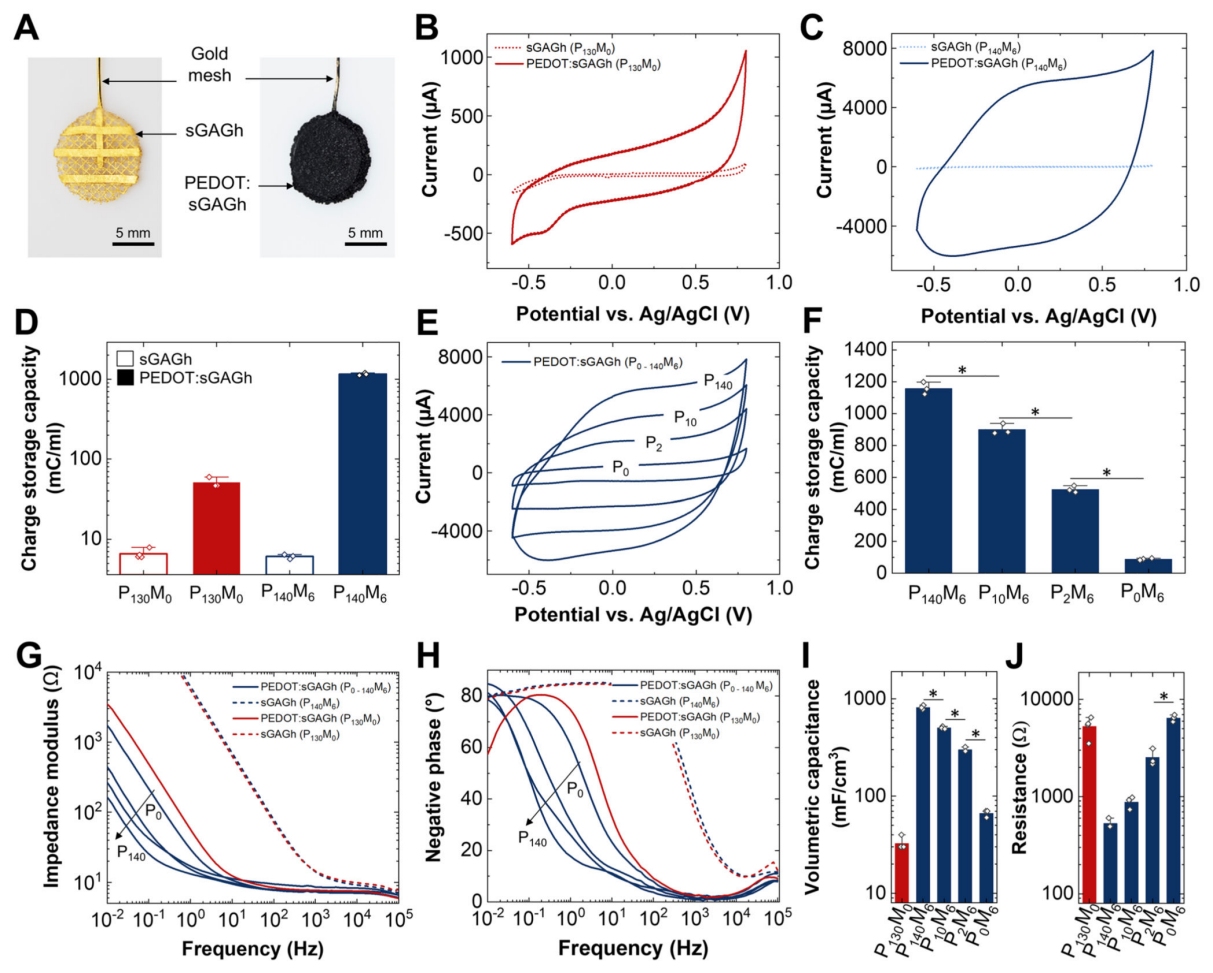
Tunability of electrochemical properties. (A) For electrochemical experiments, hydrogels (sGAGh) were integrated on gold mesh electrodes followed by polymerization of PEDOT (PEDOT:sGAGh). Hydrogels serve as working electrodes in a standard 3-electrode electrochemical cell with PBS as the electrolyte. CV measurements were performed at 50 mV/s. (B) CV of P_130_M_0_ hydrogels with/without PEDOT. (C) CV of P_140_M_6_ hydrogels with/without PEDOT. Note the different current scale from B. (D) The charge storage capacity (CSC) defined as the integral of the cathodic current observed during CV is calculated for sGAGh and PEDOT:sGAGh. (E) CV of PEDOT:sGAGh with varying anionic charge density (0–140 μmol/mL) and fixed amphiphilicity (*M* = 6). (F) CSCs calculated from the CV measurements in E. (G) Impedance modulus and corresponding (H) phase angle for sGAGh and PEDOT:sGAGh. (I) The Randles equivalent circuit is fitted to EIS spectra to extract the volumetric capacitance and (J) resistance of PEDOT:sGAGh. Bar plots (D, F I and J) show individual data points, averages and + 1 S.D. Statistical significance was assessed using Kruskal-Wallis ANOVA, followed by a Conover’s post-hoc test for multiple comparisons. Differences were considered statistically significant at *p* < 0.05 and are indicated with *.

To study the bulk electrical properties of the hydrogels we employed electrochemical methods. Hydrogels (100 μL) were formed around gold mesh electrodes (∅ = 11 mm) enabling them to be connected to a potentiostat for performing cyclic voltammetry (CV) and electrochemical impedance spectroscopy (EIS) measurements (Figure 4A). The incorporation of PEDOT has a profound impact on the electrochemical activity of both sGAGh systems (Figure 4B,C). Higher currents and charge storage capacity in excess of 1 C/mL are observed for the PEDOT:sGAGh (P_140_M_6_) formulation where percolating clusters form (Figure 4D). The lack of distinct electrochemical peaks and the ‘box’ shape of the CV curve seen in both PEDOT:sGAGh formulations are indicative of PEDOT:polyanion complexes where conductive phases are well dispersed and interconnected [45].

As a p-type material, PEDOT is doped during oxidative polymerization and the resultant polarons and bipolarons (holes) are stabilized by charge complexation with a polyanion [46]. In our PEDOT:sGAGh system, anionic charges are contributed by ionized sulfate moieties fixed on the molecular template. To better understand their contribution to bulk electrical properties, we fixed the amphiphilicity of the sGAGh template at *M* = 6 and systematically varied the anionic charge density, *P*, from 0 μmol/mL (P_0_M_6_, no heparin) up to 140 μmol/mL (P_140_M_6_). Increasing *P* within this range leads to an order of magnitude increase in electrochemical current and charge storage capacity (Figure 4E,F). To a varying degree, incorporation of PEDOT results in lower impedance for all hydrogels (Figure 4G,H). EIS data was fitted to a Randles equivalent circuit model, which allowed us to extract values for putative electronic components such as volumetric capacitance and Faradaic resistance (Figure S8). With increasing anionic charge density, the volumetric capacitance increases while the resistance decreases (Figure 4I,J). The highest volumetric capacitance exceeding 800 mF/cm^3^ is observed in the P_140_M_6_ hydrogel.

These results indicate that higher anionic charge density improves the electronic conductivity of the PEDOT network allowing for more efficient charge transfer with the electrolyte involving the entire bulk of the hydrogel. A possible explanation is that a denser arrangement of anionic charges on the sGAG template contributes to both improved mobility of polarons/bipolarons along the PEDOT backbone as well as enhanced ionic conductivity within the clusters. Interestingly, appreciable electroactivity was observed even in the absence of sGAG (P_0_M_6_). In this formulation, the presence of maleimide nucleation sites may still enable the formation of PEDOT-rich domains whose conductivity is enhanced by small mobile anions from the electrolyte. Taken together, our data indicates that both *M* and *P* influence the bulk electrical properties of PEDOT:sGAGh. While the former influences conductivity by tuning the amount of PEDOT, the latter contributes by increasing the intrinsic conductivity along polymer chains.

### 2.4 Interactions with Growth Factors are Under Electronic Control

**Figure 5:**
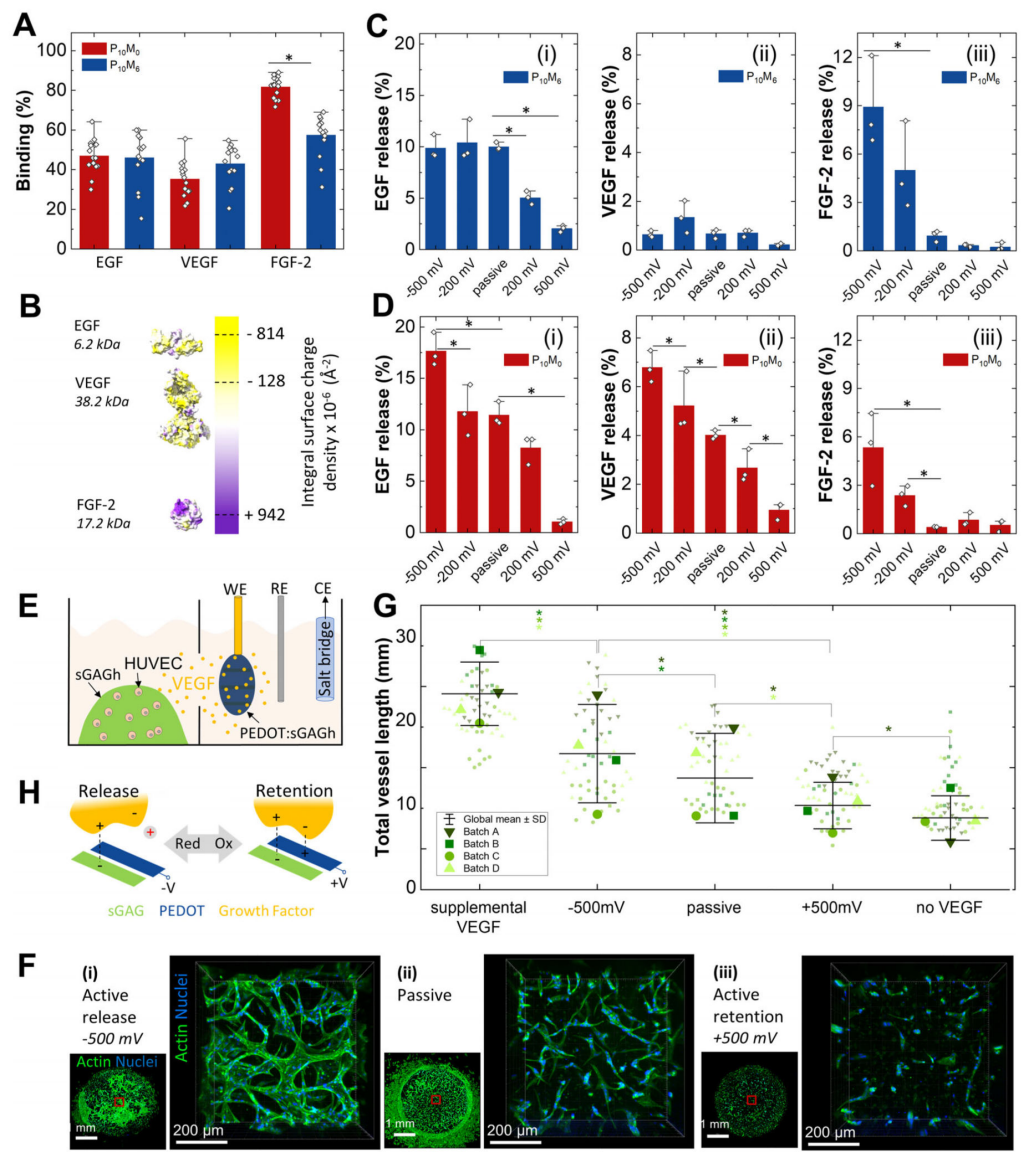
Sequestration and electrically controlled growth factor release. (A) Sequestration capacity of P_10_M_0_ and P_10_M_6_ PEDOT:sGAGh hydrogels (100 μL). The loading solution consists of 40 ng of each protein in 2.5 mL PBS. (B) Illustrative structures of human EGF, VEGF and FGF-2 with their net charge at neutral pH and molecular weight. (C) and (D) The fraction of initially loaded protein released under electrical stimulation in PBS (8 h, constant potential) and passive conditions (8 h, open circuit) for P_10_M_6_ (C) and P_10_M_0_ (D) formulations of PEDOT:sGAGh. (E) Electrochemical cell modified to decouple the effects of electrical and biochemical stimulation. HUVEC cells are embedded in sGAGh (droplets of 4 μL) with MMP-cleavable peptides as crosslinkers to allow cell-mediated matrix remodeling. The PEDOT:sGAGh working electrode, a reference electrode and a salt bridge connecting to a counter electrode are inserted in a fluidically connected compartment. (F) Representative images of HUVEC cells in sGAGh exposed to stimulated release (i), passive release (ii) and stimulated retention of VEGF (iii). Images are taken 6 days post stimulation. The actin cytoskeleton is stained with phalloidin and cell nuclei are stained with DAPI. (G) Quantification of the HUVEC filament length. The total filament length is calculated for separate fields of view (1240 × 1240 μm^2^, small symbols) covering the internal volume of sGAGh. The experiment is repeated four times (Batch A–D). Large symbols indicate batch averages, horizontal lines indicate grand averages and whiskers indicate ± 1 S.D. (H) A simplified model of electrostatic interactions governing growth factor release and retention in PEDOT:sGAGh. Bar plots (A, C and D) show individual data points, averages and + 1 S.D. Statistical significance (A, C, D and G) was assessed using Kruskal-Wallis ANOVA, followed by a Conover’s post-hoc test for multiple comparisons. Differences were considered statistically significant at *p* < 0.05 and are indicated with *.

Next, we investigated the interaction of PEDOT:sGAGh with biologically active signaling proteins. To approximate the anionic charge densities characteristic of soft tissue (e.g., *P* ≈ 15–43 μmol/mL in mammalian brain [47], we focused on P_10_M_0_ and P_10_M_6_ PEDOT:sGAGh variants. We first confirmed that permeability to macromolecules of sizes comparable to growth factors is retained following PEDOT incorporation (Figures S5 and S9). As shown in Figure 5A, PEDOT:sGAGh sequestered approximately 50% of growth factors from a loading solution containing a mixture of Epidermal Growth Factor (EGF), Vascular Endothelial Growth Factor (VEGF), and Fibroblast Growth Factor-2 (FGF-2). This cocktail represents proteins of varying size, net charge and distribution of positively and negatively charged residues (Figure 5B).

Having established the affinity of growth factors to PEDOT:sGAGh, we turned to investigating if their retention and release can be modulated by electrical stimulation. Applying electrical potentials through the gold mesh generates currents that, depending on polarity, either inject or extract holes from the PEDOT:sGAGh, thereby modulating its redox state (Figure S10). We applied oxidizing (positive) and reducing (negative) potentials (vs. Ag/AgCl) within the water window to PEDOT:sGAGh hydrogels pre-loaded with the growth factor cocktail. We then quantified the fraction of the initially loaded growth factors released into solution. These electrically stimulated release profiles were compared to passive release from hydrogels disconnected from any circuit. For both PEDOT:sGAGh variants (P_10_M_0_ and P_10_M_6_), oxidative potentials promoted retention of growth factors relative to passive release, while reducing potentials enhanced release (Figure 5C,D). Notably, the application of either potential did not induce measurable swelling or deswelling of the hydrogels (Figure S11). Control hydrogels lacking PEDOT (sGAGh) did not exhibit any electrically modulated release and showed lower passive release, likely due to a larger proportion of unscreened heparin binding sites (Figure S12).

To confirm that growth factors remain biologically active, we assessed the effect of VEGF release in a vasculogenesis model using human umbilical vein endothelial cells (HUVECs) embedded in sGAGh [48]. An electrochemical setup was designed in which the HUVEC compartment was fluidically connected to the PEDOT:sGAGh (P_10_M_0_) compartment, but electrically isolated from the ionic currents created during growth factor release. This ensures that cell response was due to VEGF and not to electrical stimulation (Figure 5E). VEGF loading was adjusted so that passive release would approach the threshold required to trigger a cellular response (Figure S13). PEDOT:sGAGh was subjected to a reducing potential (–500 mV), no potential, or an oxidizing potential (+500 mV) for 3 h, resulting in estimated VEGF release doses of 129, 63, and 13 ng, respectively. In cultures exposed to the released VEGF, HUVECs formed tubular structures resembling early-stage vasculogenesis after 6 days of culture (Figure 5F). These structures were most prominent under reducing conditions, which enhanced VEGF release, and were nearly absent under oxidizing conditions, where VEGF was retained (Figure 5G; Figure S14). Additional control experiments confirmed that applying a reducing potential to PEDOT:sGAGh without preloaded VEGF did not induce tubule formation, while supplementing exogenous VEGF during oxidizing potentials partially restored the vasculogenic response (Figure S15).

Our findings suggest that the affinity of growth factors for PEDOT:sGAGh is primarily governed by electrostatic interactions. A possible explanation is that charge complexation between sulfate groups in sGAG and polarons in PEDOT generates localized dipoles, which in turn mediate interactions with charged residues on protein surfaces (Figure 5H). When oxidative potentials are applied, hole injection into PEDOT increases the population of positive charges. In combination with the negative charges of the sGAG component, the dipolar landscape is thus enhanced. This in turn increases the electrostatic interactions with charged protein residues (present in a wide variety of proteins with varying net charges). Conversely, applying reducing potentials withdraws hole charges, destabilizing the dipolar interactions and facilitating the release of bound proteins into solution.

### 2.5 PEDOT:sGAGh is Integrated in Devices and Circuits

**Figure 6:**
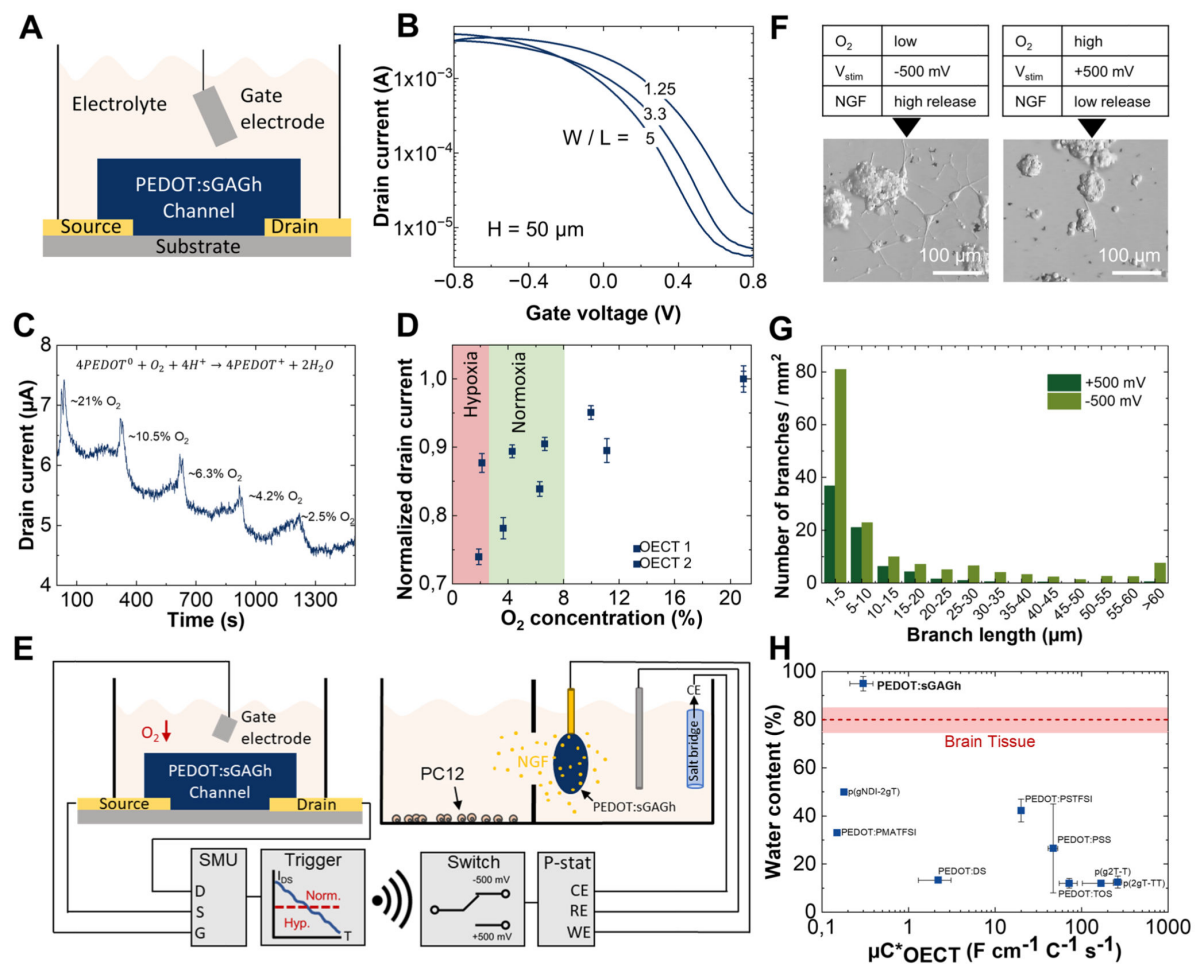
Molecular sensing and actuation enabled by PEDOT:sGAGh devices. (A) Schematic of an OECT with a channel formed from PEDOT:sGAGh (P_140_M_6_). The channel is contacted by gold source and drain electrodes and gated by an Ag/AgCl electrode. (B) Transfer curves of OECTs with different channel aspect ratios (width/length) and constant height (H = 50 μm). (C) Modulation of the drain current (*I_SD_*) by the amount of dissolved oxygen (% of dissolved gasses). Reference oxygen concentration measurements are provided by a commercial sensor. Inset: O_2_ reduction at the channel leads to enhanced doping of PEDOT in PEDOT:sGAGh. (D) Typical sensor responses for physiologically relevant oxygen concentrations. Error bars at each O_2_ concentration represent the average and ± 1 S.D. of *I*_SD_ at steady state. (E) Biohybrid circuit combining O_2_ sensing and NGF release. A source measurement unit (SMU) interfaces the OECT sensor with a computer where *I*_SD_ is compared to a preset threshold. An ON/OFF signal is wirelessly transmitted to another computer which instructs a potentiostat to apply ±500 mV to a PEDOT:sGAGh electrode loaded with NGF. (F) Representative images of live PC12 cells (5 days after stimulation) following exposure to O_2_ concentration modulated electrical release of NGF. Neurite-like structures are formed in cultures exposed to high levels of NGF. (G) Histogram quantifying the number density of neurites grown following active release or retention of NGF. The number of branches is the sum from 6 fields of view (each 1.1 mm^2^) from each condition. (H) Water content and μC*_[OECT]_ comparison of PEDOT:sGAGh with previously reported PEDOT-based OECT channel materials [59–62]. Data points represent the average of μC*_[OECT]_ and water content and error bars indicate ± 1 S.D.

We integrated PEDOT:sGAGh as the active channel material in organic electrochemical transistors (OECTs) by combining microfluidic molding of sGAGh with subsequent oxidative polymerization of PEDOT (Figure 2F(ii), Figure 6A; Figure S16). We incorporated the formulation with the highest electroactivity (P_140_M_6_) and tested its switching behavior in PBS (Figure 6B). The resulting OECTs exhibited source-drain current (*I_SD_*) modulation over three orders of magnitude at gate voltages constrained to the water window. To build upon the multimodal functionality of PEDOT:sGAGh, we demonstrated that our OECTs can function as sensors by measuring oxygen concentrations in PBS (Figure 6C). This enabled us to monitor physiologically relevant oxygenation levels (Figure 6D) and to build a biohybrid circuit emulating the adaptive responses of hypoxic brain tissue (Figure 6E) [49–51]. In this system, the detection of low oxygen triggers the application of reducing potential (−500 mV, 3 h) to release nerve growth factor (NGF) from PEDOT:sGAGh. The liberated NGF stimulates neurite outgrowth in a PC12 cell model of neuronal differentiation observed 5 days after the stimulation event (Figure 6F,G; Figure S17).

Our results demonstrate that PEDOT:sGAGh is an effective channel material in OECT devices. A key figure of merit for channel performance is the product of charge carrier mobility (μ) and volumetric capacitance (C*), known as μC*. For PEDOT:sGAGh (P_140_M_6_), we measured a μC* value of 300 ± 90 mF/(cm⋅V⋅s) which is comparable to values reported for other polysaccharide-doped channel materials [52]. While OECTs have had much success integrating PEDOT-based thin films, these devices typically struggle to match the hydration state and softness of tissue (Figure 6H) [53]. The elevated water content may make PEDOT:sGAGh-based OECTs well-suited for mimicking the mechanical properties of tissues while enabling sensing of biomolecular signals that evolve over timescales of seconds to minutes [54]. In our proof-of-concept biohybrid circuit, the electrocatalytic activity of PEDOT enables oxygen reduction to water at gate voltages within the electrochemical water window [55]. Under a positive gate bias (*V_GS_* = + 400 mV), this redox reaction increases hole injection and channel doping, thereby enhancing device conductance [56]. While these measurements were conducted in the absence of interfering electrochemical side reactions, oxygen specificity could be further improved for future implanted applications by incorporating oxygen-selective membranes in the sensor design [57, 58].

## 3 Discussion

Our work presents in situ polymerization of an organic semi-conductive polymer within an ECM-inspired hydrogel template, yielding a material that is bioactive, electrochemically responsive, and dynamically addressable. We find that hydrophobic substitutions in the hydrogel template likely play an important role in evolving conductive nanoarchitectures by promoting the formation of PEDOT-rich clusters. As a result, PEDOT:sGAGh structurally resembles 3D filled polymer composites, where electronic charge is transported through percolating networks of conductive particles [63]. However, unlike conventional filled elastomers where the binding matrix is a dielectric elastomer, PEDOT:sGAGh consists predominantly of water (≈95 wt.%). This high-water content enables access and loading of macro-molecules such as proteins within the 3D bulk of the material. In contrast, devices relying on thin-film materials (PEDOT:PSS), despite their mechanical compliance enable only surface interactions with proteins. Another defining feature of our material system is the exceptionally low PEDOT content required to confer electroactivity. Incorporating less than 1 wt.% of PEDOT is sufficient to transform a non-electroactive sGAGh into an electrically addressable biomaterial. Incorporation of PEDOT preserves the high-water content and tissue-like elastic modulus of PEDOT:sGAGh. While the resulting volumetric capacitance (≈800 mF/cm^3^), charge storage capacity (≈1 C/mL), and μC* (≈300 mF/(cm⋅V⋅s)) are modest compared to those of PEDOT:PSS films or hydrogels designed for supercapacitors, these values must be interpreted in light of material composition, softness and hydration levels [52, 59, 60]. In contrast to other conductive hydrogels—which typically contain 5–50 wt.% semiconductive polymer [29, 64–66]—our system achieves functional conductivity with a fraction of that content, which likely contributes to preservation of the bioactivity of the sGAGh template. While PEDOT is generally considered well-tolerated by biological systems, reducing the required amount may contribute to lower oxidative stress and immune responses in tissues [67]. Thus, our demonstrations of sensing and actuation using PEDOT:sGAGh suggest that high electrical performance is not necessarily a prerequisite for achieving meaningful bioelectronic functions.

Our choice of template is inspired by the critical role that sGAGs play in the function of ECMs. In native ECM, the electrostatic environment, shaped by the distribution of fixed anionic charges, is a key regulator of the availability, spatial distribution, and bioactivity of growth factors and cytokines [68, 69]. The spatiotemporally controlled release or sequestration of these signaling molecules depends on enzymatic degradation and synthesis of sGAG networks. In contrast, electronically active biomaterials such as PEDOT:sGAGh may offer a distinct advantage because they enable dynamic, on-demand control over biomolecule release without requiring structural remodeling of the underlying macromolecular matrix, which is a functional capability absent in native ECM.

Other bioartificial tissue strategies rely on optical or mechanical stimuli for delivery of extracellular signaling proteins [70, 71]. In contrast to established delivery systems, the advantage of our approach is that it provides a direct interface between electronic and biological systems. We further demonstrated that the same material platform can be used for sensing molecularly encoded information by coupling it to an electronic readout, thus enabling the possibility of bidirectional communication using a limited set of materials. Our work represents an important first step toward creating an electronic extracellular matrix (eECM). However, several challenges remain before fully functional eECMs can be realized. For therapeutic applications, it is essential to maintain proteins in their bioactive form for clinically relevant periods of time. Achieving this would enable pulsed, on-demand electrical modulation of anti-inflammatory and pro-regenerative factor availability. Although we did not specifically evaluate pulsed and long-term stimulated retention and release beyond several hours, our material has the potential to sequester proteins from surrounding cells, thereby replenishing the local reservoir of bioactive molecules. Realizing this will depend on designing eECMs with tunable affinity for defined sets of target proteins.

Future designs may need to incorporate additional abundant ECM components such as fibrous proteins (e.g., collagen, elastin) and glycoproteins (e.g., fibronectin, laminin), which alongside proteoglycans, play central roles in regulating cell interactions. A robust eECM must also be capable of encapsulating cells, and therefore must be remodelable. Its electrically controlled functions must be molecularly and cell-type specific, and should operate orthogonally to the effects of conventional electrical stimulation.

Our conductive hydrogel platform offers a promising approach for bridging biomolecular signaling in living tissues with the computational capabilities of solid-state electronics. This convergence opens the door to bioelectronic interfaces that go beyond conventional modalities like electrical recording and neuromodulation. By enabling electronically controlled delivery of regenerative and reparative molecular cues, such systems could evolve from mere neuroprosthetics into active agents of tissue repair that are capable of addressing damage caused by injury or disease.

## 4 Methods

### 4.1 Preparation of PEDOT:sGAGh

#### 4.1.1 Preparation of Maleimide-Conjugated Heparin

Maleimide-conjugated heparin was synthesized in-house. Heparin (Merck, 375095–500KU, apparent *M*_n_ ≈ 11 500 g/mol, Ð ≈ 1.55, 1000 mg, ≈0.0015 mol COO^−^, 1 equiv) was dissolved at 4°C in 10.8 mL ice-cold Milli Q water in a glass vial equipped with a magnetic stir bar and cooled in an ice bath. First the sNHS (N-Hydroxysulfosuccinimide sodium salt, Sigma-Aldrich, 56485, 173.7 mg, 0.53 equiv) and then the EDC (N-Ethyl-N’-(3-dimethylaminopropyl)carbodiimide hydrochloride, IRIS Biotech, RL-1022, 347.4 mg, 1.07 equiv) were dissolved in ice-cold Milli Q water (1.6 and 0.8 mL respectively) and added sequentially (first sNHS, then EDC once the sNHS was fully dissolved) to the stirred heparin solution. The Heparin/sNHS/EDC solution was stirred for 20 min to activate the carboxyl groups. After 20 min an ice-cold solution of MalTFA (N-(2-Aminoethyl) maleimide trifluoroacetic acid salt, Sigma-Aldrich, 56951, 139.0 mg, 0.43 equiv) in 0.8 mL Milli Q water was slowly added dropwise to the activated heparin solution. After a further 10 min, the ice bath was removed and the reaction was allowed to stir for 3 h. The reaction solution was then transferred to dialysis tubing (MWCO = 2 kDa, 29 mm diameter, Spectra/Por Prewetted RC Tubing) and dialyzed against 1.0 M NaCl (176 g NaCl in 3.0 L deionized water) overnight. The dialysis bath was subsequently changed each morning and evening for 1 × 1.0 M NaCl, and 3x Milli Q water over 2 days. The product was then frozen and lyophilized.

#### 4.1.2 sGAGh Template Hydrogels

Thiol-maleimide hydrogel templates (P_0-140_M_6_) were prepared by employing Michael-type addition click chemistry. 4-arm thiol terminated polyethylene glycol (starPEG-SH, *M*_n_ ≈ 10 000 g/mol, JenKem Technology) and maleimide conjugated heparin (*M*_n_ ≈ 11 500 g/mol) were mixed with the molar ratio between PEG-SH and heparin maleimide molecules kept at 1. Their reaction forms covalent thiosuccinimide crosslinks. Maleimide-terminated 4-arm polyethylene glycol (PEG-mal, *M*_n_ ≈ 10 000 g/mol, JenKem Technology) was used to partly or fully replace heparin maleimide to control the anionic charge density of the hydrogel (*P*), while keeping the solid content and crosslinking degree constant. All precursors were dissolved in diluted (0.01 x) PBS (Sigma-Aldrich) with the pH adjusted to achieve a gelation time between 15 s and 5 min. This was confirmed by mixing the precursor solutions under continuous pipetting until gelation was verified visually [72].

EDC/NHS crosslinked hydrogel templates (P_10/130_M_0_) were prepared by mixing 4-arm PEG-NH_2_ (*M*_n_ ≈ 10 000 g/mol, JenKem Technology) and heparin at a molar ratio of PEG to heparin molecules equal to 1. To tune the integral charge density (*P*), of the hydrogel, 4-arm PEG-COOH (*M*_n_ ≈ 10 000 g/mol, JenKem Technology) was used to replace a fraction of the heparin. EDC and NHS were used to activate the carboxyl group of heparin molecules or PEG-COOH for crosslinking with the primary amines from PEG-NH_2_, forming a covalent amide bond. Gelation was verified visually, occurring in approximately 3 h as reported previously [73]. The higher swelling ratio observed in EDC/NHS hydrogels was compensated by adjusting the solid content so that the final charge density (*P*), could be kept within nearly the same range as in the thiol-maleimide system. The composition of all types of sGAGh templates produced is listed in Table S1.

#### 4.1.3 Preparation of PEDOT:sGAGh by Oxidative Polymerization

Oxidative polymerization was employed to incorporate PEDOT into the hydrogel according to previously published protocols [22]. Briefly, the formed sGAGh templates were incubated in a 0.4 M ammonium persulfate solution (APS, Carl-Roth) dissolved in 1 M hydrochloric acid (HCl, Carl-Roth) for 3 h. Afterward, the hydrogels were immersed in 0.4 M 3,4-ethylenedioxythiophene (EDOT) solution (Sigma-Aldrich) dissolved in mineral oil (Sigma-Aldrich) for 6 h. After the incubation, the remaining monomer and oil were removed by gently washing in hexane (Honey-well) followed by washing overnight and storage in PBS prior to use.

#### 4.1.4 Preparation of Electrode-Integrated PEDOT:sGAGh Samples

Hydrogels were prepared by placing a 11 mm diameter gold mesh electrode (Fiaxell, gold M Grid) on a Sigmacote (Sigma-Aldrich) treated, hydrophobic glass coverslip (12 mm diameter). 100 μL of sGAGh precursor solution was added and covered by a second hydrophobic cover slip with the same diameter. After 30 min the samples were swollen in PBS and the coverslips were removed. Incorporation of PEDOT follows the procedure described above.

### 4.2 Characterization of PEDOT:sGAGh

#### 4.2.1 Small-Angle x-Ray Scattering (SAXS)

SAXS data were collected with a Bruker Nanostar equipped with a 2D Vantec-2000 area detector and a microfocus x-ray source (IμS), operated at 50 kV and 0.6 mA. The x-ray beam had a wavelength of 1.54 Å (Cu K*α*) and a focal spot size of 115 μm. The sample to detector distance was 107.7 cm, as calibrated with silver behenate scattering. Each specimen was measured in dry conditions at three different locations within the specimen. SAXS data were corrected for background and radially averaged.

#### 4.2.2 Transmission Electron Microscopy (TEM)

sGAGh and PEDOT:sGAGh samples were dissected into 1–3 mm small pieces, washed in water and *en bloc* contrasted with 1% aqueous uranyl acetate (UA, Polysciences, 21447) overnight at 4°C [69, 70]. Samples were then washed in distilled water (5x for 5 min), followed by dehydration in a graded series of ethanol/water solutions up to pure ethanol (30%, 50% for 20 min each; 70%, 90%, 96% for 30 min each; 3 × 100% ethanol on molecular sieve for, 30 min each). This was followed by infiltration with the epon substitute Embed 812 (an Epon 812 replacement) using resin/ethanol mixtures (resin:ethanol at 1:3, 1:1, and 3:1 for 1 h each, followed by an incubation overnight and for another 5 h in pure epoxy resin). Finally, the samples were placed on top of an empty resin dummy block and excess of epoxy resin was removed with a filter paper. Samples were cured at 65°C overnight, and ultrathin sections (70 nm) were prepared with a Leica UC6 ultramicrotome (Leica Microsystems), collected on formvar-coated slots or 300 mesh grids, and contrasted with uranyl acetate. Contrasted sections were analyzed on a Jeol JEM-1400 Plus (Jeol) running at 80 kV acceleration voltage [74, 75]. The particle area and fill fraction of the UA-stained heparin molecule were analyzed using ImageJ software. After background subtraction, a threshold (0–200) was applied. The ‘watershed’ tool was used to separate adjacent peaks followed by the application of the ‘analyze particles’ tool.

#### 4.2.3 Conductive Atomic Force Microscopy (cAFM)

sGAGh and PEDOT:sGAGh samples were processed as described in the previous section (TEM) cAFM was performed using a Dimension Icon AFM (Bruker) with 0.5 Hz scan rate and 480 × 480 pixel resolution in PeakForce TUNA mode with 1 kHz oscillations to reduce adverse lateral shear forces. The current was detected during tip-sample contact via a current amplifier with a sensitivity of 100 pA/V. Each measurement series of the samples was performed in ambient conditions using the same respective TUNA Peakforce cantilever (Bruker AFM Probes; nominal spring constant = 0.4 N/m) to allow for qualitative comparison between compositions at voltages of −30, −20, 20 and 30 mV, after subtraction of the instrument-specific offset of −7.5 mV. Tip conductivity was ensured via cAFM measurements on a test sample before and after each series. The particle area and fill fraction of the conductive clusters were analyzed using ImageJ software after application of a four-pixel Gaussian filter using Gwyddion software. After background subtraction an automatic threshold was applied. The ‘watershed’ tool was used to separate adjacent peaks followed by the application of the ‘analyze particles’ tool.

#### 4.2.4 Atomic Force Microscopy (AFM)

The mechanical properties of hydrogels were quantified using a Nanowizard IV AFM (Bruker) integrated with an inverted Axio Observer A1 optical microscope (Zeiss). Samples were measured at room temperature while submerged in PBS within a temperature-stabilized sample chamber (Petri-Dish Heater; Bruker). For indentation measurements, tipless silicon nitride cantilevers (PNP-TR-TL-Au, Nanoworld) with a nominal spring constant of 80 mN m^−1^ were modified with 10 μm silica beads (Kisker Biotec GmbH) and calibrated using the thermal noise method [76]. Force-displacement curves were recorded in closed-loop, constant height mode at an approach and retraction velocity of 5 μm/s with a relative force setpoint of 3 nN. A minimum of 20 locations were probed on each sample, with three independent replicates analyzed for each condition. The resulting force curves were analyzed using the AFM manufacturer’s software (Bruker) to extract the elastic modulus (*E*) by fitting the Hertz model to the approach data [77].

#### 4.2.5 Gravimetric Compositional Analysis

Identical freestanding samples of sGAGh (*n* = 8) were prepared as described previously. Half of the samples (*n* = 4) were functionalized with PEDOT to form PEDOT:sGAGh. All samples were then washed in Milli Q water for one day to remove residual salts. The hydrogels were then dried at 60 °C overnight before their mass was measured. The mass of each dry PEDOT:sGAGh sample is normalized to the average dry mass of sGAGh hydrogels (*n* = 4).

#### 4.2.6 Electrochemical Characterization

Cyclic voltammetry (CV) and Electrochemical impedance spectroscopy (EIS) were performed using a MultiPalmSens4 or PalmSens4 (PalmSens) potentiostat. We used a three-electrode setup with a Ag/AgCl reference electrode (Metrohm, 6.0726.100), a carbon mesh counter electrode (BioLogic, A-010530) and a gold mesh integrated sGAGh or PEDOT:sGAGh as the working electrode. All electrodes were immersed in 1 × PBS as the elec-trolyte. For CV measurements, the electric potential between the working electrode and the reference was scanned from −0.6–0.8 V vs. Ag/AgCl at 50 mV/s and the current between the working and the counter electrode was measured. EIS was conducted using the same electrode and electrolyte setup using an excitation amplitude of 10 mV_RMS_ applied within the frequency range of 0.01 Hz to 100 kHz. Equivalent circuit fitting was performed using MultiTrace software (PalmSens) based on the Randles circuit, which includes a series resistor (electrolyte resistance) and a parallel combination of a resistor (charge transfer resistance) and a capacitor (volumetric capacitance).

### 4.3 PEDOT:sGAGh Interaction with Proteins

#### 4.3.1 Protein Uptake and Electrically Controlled Protein Release

A loading solution of Epidermal Growth Factor (human EGF – recombinant protein, PeproTech), Vascular Endothelial Growth Factor (human VEGF – 165 recombinant protein, PeproTech), and Fibroblast Growth Factor 2 (human FGF-2-basic – 154aa recombinant protein, PeproTech) was prepared by dissolving 40 ng/mL of each protein in 2.5 mL of PBS supplemented with 0.1 *m/v* % Bovine Serum Albumin (BSA, Sigma-Aldrich, A7030) and 0.1 *v/v* % biocide ProClin 300 (Sigma-Aldrich). Hydrogels (sGAGh and PEDOT:sGAGh integrated on gold meshes as described before) were incubated overnight in the loading solution in non-binding tubes to load them with proteins. Uptake fractions were calculated by measuring the remaining protein in the loading solution and subtracting it from the initial protein concentration.

For electrically controlled protein release experiments, we employed a modified 3-electrode electrochemical cell. Pre-loaded PEDOT:sGAGh hydrogels were placed in a tube filled with 5 mL PBS supplemented with 0.1 *m/v* % BSA and 0.1 *v/v* % biocide ProClin 300 together with a Ag/AgCl (DRIREF-2, Dri-Ref WPI) or Ag/AgCl-wire reference electrode. A carbon mesh was placed in a separate beaker filled with PBS and used as a counter electrode. The two compartments were connected with a salt bridge consisting of a polyacrylamide/agarose filled silicone tube, of which one end was sealed by a dialysis membrane (mesh size < 1 kDa) to prevent proteins from entering the salt bridge. Loaded PEDOT:sGAGh were transferred to the release chamber and different electrical potentials (vs. the Ag/AgCl reference) were applied for 8 h using a MultiPalmSens4 potentiostat. Enzyme-linked Immunosorbent Assays (R&D Systems, human EGF DuoSet ELISA DY236, human FGF basic DuoSet ELISA DY233, human VEGF DuoSet ELISA DY293B) were used to quantify protein concentrations in the PBS sample following the instructions given by the supplier.

For experiments involving cell cultures, we added another chamber (10 mL) housing either HUVEC or PC-12 cells. The cell culture chamber was connected to the main protein release chamber by a circular port (3–4 mm). This ensures electrical currents are confined in the release chamber, thus decoupling the effects of electrical stimulation from those of growth factor release. For experiments with cells, we optimized the protein loading so that the passively released dose would be around the threshold needed to differentiate cells (Figures S13 and S17). For experiments with HUVECs, we loaded PEDOT:sGAGh (P_10_M_0_) with protein by incubation in 2.5 mL of PBS containing 10 μg of VEGF-165, 0.1 *m/v* % BSA and 0.1 *v/v* % Antibiotic Antimycotic Solution (Sigma-Aldrich). For experiments with PC12, we loaded PEDOT:sGAGh (P_10_M_0_) by incubation in 2.5 mL of PBS containing 1 μg of rat NGF-*β* (Sigma Aldrich, N2513) and 0.1% BSA. Growth factor loaded hydrogels were washed for 1 h in 2.5 mL PBS with 0.1 *m/v* % BSA to remove loosely bound proteins. For electrically triggered release experiments, cell culture media was supplemented with 5 μg/mL catalase (Sigma-Aldrich) as an antioxidant to counter the effects of electrochemically produced hydrogen peroxide. Subsequently, 3 h of electrical stimulation was applied to the working electrode (−500 mV for release, +500 mV for retention) with a multichannel MultiPalmSens4 potentiostat. After electrical stimulation, cell culture media between two chambers were mixed and all electrodes were removed. To obtain positive controls (supplemental VEGF condition in Figure 5G; Figures S14 and S15) we stimulated HUVEC cells with 500 ng of VEGF in 10 mL media.

#### 4.3.2 Visualization of Protein Structures

The protein structures in Figure 5B are provided for illustrative purposes and are sourced from the Worldwide Protein Data Bank (wwPDB) as follows: Human epidermal growth factor (https://doi.org/10.2210/pdb1JL9/pdb), Human basic fibroblast growth factor (https://doi.org/10.2210/pdb2FGF/pdb), and Human vascular endothelial growth factor (https://doi.org/10.2210/pdb1VPF/pdb). To visualize charged residues, structures were imported into a molecular visualization program (ChimeraX, UC San Francisco) and the ‘coulombic’ command is used to visualize the Coulombic electrostatic potential of the molecule. Net charge at pH 7.4 is calculated in Prot Pi (Center for Biochemistry and Bioanalytics of Zurich University of Applied Science) using the peptide sequence provided by the growth factor manufacturer (PeproTech).

### 4.4 Cell Cultures

#### 4.4.1 Human Umbilical Vein Endothelial Cells (HUVECs)

HUVECs labeled with green fluorescent protein (GFP) were purchased from Angio-Proteomie and expanded in Endothelial cell growth medium (Promocell) according to the supplier instructions. Briefly, 75 cm^2^ cell culture flasks (Thermo Scientific Nunc EasYFlask) were coated by adding 5 mL of a 20 μg/mL fibronectin solution. The solution was incubated for 30 min at 37°C and 60 min at room temperature. After aspiration of the fibronectin solution, cells were seeded and expanded in Endothelial cell growth medium with SupplementMix (C-39215, Promocell) and 1% penicillin/streptomycin (Capricorn Scientific). The cells were maintained at 5% CO_2_ and 37°C. The HUVECs were detached using trypsin-ethylenediaminetetraacetic acid (0.5%, Sigma-Aldrich) after reaching 80% confluency before embedding in sGAGh matrix.

HUVECs (harvested from passage 9 or 10) were embedded in an enzyme degradable version of the sGAGh hydrogel at a cell concentration of 1 × 10^6^ cells/mL. The hydrogel formulation and embedding procedure follow a previously described method with slight modifications [48]. Briefly, the hydrogel was formed from 4-arm starPEG-MMP-SH (*M*_n_ ≈ 15 000 g/mol, 0.92 mM molecules, MMP cleavable sequence: GCGGPQG↓IWGQGGCG), heparin conjugated with maleimide (*M*_n_ ≈ 15 000, 0.62 mM), and a cell-adhesive peptide (CWGGRGDSP, RGD; *M*_n_ ≈ 990 g/mol, 1 mM) synthetized in house (Figure S18).

To prepare hydrogels, starPEG-MMP-SH and heparin-maleimide were dissolved separately in 1x PBS (pH 7.4). The adhesive peptide was added to the heparin solution followed by addition of the cells suspended in the heparin-maleimide precursor solution. An equal volume of starPEG-MMP-SH solution was quickly combined with the cell-heparin mixture, then added to a well in a 6-well adhesion plate (Techno Plastic Products, TPP 92406). The gelation time was tuned by adding 0.1 M HCl to the starPEG-MMP-SH precursor solution until reaching the range of 30–60 s. The amount of HCl added varied depending on the precursor batch in the range of 1–5 *v/v* %. An array of 4 × 4 μL cell-laden hydrogel droplets was formed on the bottom of the cell culture chamber. After 15 min, 5 mL of Endothelial cell growth medium with SupplementMix and 1% penicillin/streptomycin were added. Fresh 10 mL cell culture medium was added on the day of stimulation. Otherwise, 5 mL of media was replaced every 2–3 days.

#### 4.4.2 PC12 Cell Line

PC12 cells were purchased from ATCC. Cells were seeded on a collagen I (rat tail (Gibco, A10483-01) coated T25 cell culture flask in RPMI media (Gibco, 11875093) supplemented with 10% horse serum (Gibco, 26050070), 5% fetal bovine serum (Gibco, 16140063), and 1% Penicillin/Streptomycin (Gibco, 10378016). The medium was changed every 2–3 days while cells were maintained at 37°C and 5% CO_2_. Cells from passages 9 to 15 were used for the biohybrid circuit experiments. After reaching 80%–90% confluency cells were reseeded in the (collagen I pretreated) cell culture chamber of the electrochemical setup at 3 × 10^4^ cells/cm^2^.

#### 4.4.3 Cell Imaging and Analysis

Living GFP-labeled HUVEC cultures were imaged 7 days after embedding cells into hydrogels (6 days after stimulation with VEGF) with a Dragonfly Spinning Disc confocal microscope (Andor). For each sGAGh droplet, a 4 × 4 tiled image set was acquired using 10x air objective, spanning a depth of 100 μm thick images and sampled at 5 μm intervals along z-axis. Tiles were included in the analysis if their entire field of view was within the hydrogel interior and no signs of hydrogel decomposition were observed. The inverted microscope Eclipse Ts2R (Nikon) was used to acquire images. Images of the living PC12 cells were taken with a 10x air objective in brightfield using NIS-Elements (Nikon) software.

For fixation, HUVEC 3D cultures were first washed with PBS. Cultures were then fixed by 2% paraformaldehyde (Thermo scientific) in PBS for 5 min at 4°C. Cultures were then washed 3 times with PBS followed by permeabilization with 0.1% Triton X-100 for 10 min at room temperature. After washing with PBS, cultures were incubated with ATTO633-phalloidin (1:200 dilution, ATTO-TEC) and DAPI (1:10,000 dilution D8417, from 1 mg/mL in Milli Q, Sigma-Aldrich) for 3 days at 4°C in PBS with 0.1% BSA. After washing twice with PBS/0.1% BSA, samples were stored in PBS at 4°C until the day of imaging.

Custom macros in ImageJ were used to perform image analysis [48]. These included maximum intensity projection of Z-stacks, 5% contrast enhancement, gaussian blur (sigma 3 pixels), and Li thresholding for conversion to binary image. Built-in features in ImageJ “analyze particles” and “2D skeletonize” were used to quantify the length, area, and number of branching points in vessel-like HUVEC structures as previously described [55]. A similar pipeline was applied to PC12 cells to extract neurite length. Image analysis included a contrast enhancement of 40%, a background subtraction and Li thresholding for conversion to binary images. The quantification included the built-in-features “analyze particles” and “2D skeletonize”.

### 4.5 OECTs and the Biohybrid Circuit

#### 4.5.1 OECT Fabrication

Source (S) and Drain (D) contacts were defined on glass or polyimide substrates from Cr/Au (3/60 nm) films using standard lithography and etching processes. Before passivation, the metallized substrates were cleaned in a water, hydrogen peroxide, ammonium hydroxide mixture (5:1:1) for 13 min at 70°C. After a washing step in water, the samples were dried at 120°C for 30 min. For passivation of the interconnects, a 4 μm thick SU-8 (3005, micro resist technology, Kayaku) layer was patterned using standard protocols. To fabricate PEDOT:sGAGh channels with well-defined width (W), length (L) and thickness (H), a microfluidic molding approach was used. We created a soft negative mold of the OECT channels by casting PDMS (SYLGARD 184, Dow) on a Si wafer containing SU8-2050 (Kayaku) positive features. The PDMS mold was then functionalized with a highly hydrophobic silane. To achieve this, the PDMS mold was treated with air plasma for 1 min. Afterward, the mold was incubated for 10 min in a 0.5% (Tridecafluoro-1,1,2,2-tetrahydrooctyl)-1-trichlorosilane (abcr) solution in Novec 7500 (Iolitec) and subsequently washed with Novec 7500 (Iolitec) for another 10 min followed by a heating at 110°C for 10 min. For hydrogel molding, the hydrophobic PDMS mold was clamped onto the electrode chip and filled with sGAGh precursor solution by vacuum-assisted infiltration. After gelation, the mold was removed and the hydrogel was incubated in 0.1 M sodium hydroxide to assure complete cross linking of the sGAGh template. After swelling and washing in PBS, the sub-strate with the S/D contacts and the integrated sGAGh channel was immersed in solutions to form PEDOT:sGAGh (P_140_M_6_) as described before (0.4 M APS for 1 h and 0.4 M EDOT for 6 h). OECTs were characterized in PBS using a suspended Ag/AgCl electrode (DRIREF-2, Dri-Ref WPI) as the gate electrode. For the calculation of μC*_[OECT]_, transistors of 3 different channel geometries (W x L) were fabricated: 2.0 mm x 1.6 mm, 2.0 mm x 0.6 mm, 3.0 mm x 0.6 mm.

#### 4.5.2 Calculation of μC*

The product of charge carrier mobility and volumetric capacitance (μC*) was calculated using Bernard’s model in Equation 1 [78].

(1) $$g_{\text{m}}\equiv \frac{W\cdot H}{L}\mu C^{*}(V_{th}-V_{gs})$$

here, *g_m_* is the transconductance, and *W, L* and *H* are the channel width, length and height (thickness) respectively. *V_th_* is the threshold voltage and *V*_gs_ is the gate voltage. μC* is the slope from a plot of maximal transconductance *g_m_* vs. *W* ⋅ *H* ⋅ *L*^−1^ ⋅ |*V_th_* − *V*_gs_|. The maximum transconductance *g_m_* (and the associated *V*_gs_) is calculated as the maximum derivative of the absolute value of the drain current vs. gate voltage, obtained from the transfer curve of each device. OECTs with 3 different geometries were prepared to obtain variation in *W* ⋅ *H* ⋅ *L*^−1^. The threshold voltage for each device is determined from transfer curves by plotting $\sqrt{I_{\text{SD}}}$ vs. *V_gs_*. The threshold voltage is approximated as the point where the linear regime of this curve crosses the x-axis.

#### 4.5.3 Oxygen Sensing

Oxygen depleted PBS was prepared by purging with nitrogen gas. Ground truth oxygen concentration was obtained using a calibrated commercial oxygen probe (OXY-4 mini, PreSens). Oxygen depleted PBS was then used as electrolyte for OECT sensors. OECTs were operated with gate potential of +0.4 V and a drain potential of −0.5 V with an Ag/AgCl gate electrode (DRIREF-2, Dri-Ref WPI). The drain current was continuously measured and correlated to the ground truth concentration measurements. For the OECT measurement, a stable current was reached 50 s after the measurement solution was added. The measured data points were averaged and plotted with their standard deviations.

#### 4.5.4 Biohybrid Circuit

To connect oxygen sensing to NGF release from PEDOT:sGAGh, we implemented a wireless communication protocol. In short, a custom Python code allowed communication between a Source Measure Unit (Keithley 2604B) operating the OECT and a potentiostat (EmStat4S, PalmSens) operating the PEDOT:sGAGh electrode. The default setting of the system was to apply VEGF retention (+500 mV) until the OECT drain current crossed a pre-defined threshold (0.2 μA below baseline) indicating low oxygen.

When low oxygen was detected, the potentiostat applied the release potential (−500 mV). The low oxygen effect was triggered by exchanging PBS (the OECT electrolyte) with nitrogen purged PBS. The release experiment lasted 3 h, after which the electrodes were withdrawn. Without further media changes, the live PC12 cultures were imaged 5 days later.

### 4.6 Statistical Analysis

Individual data points from each experimental replicate are presented in the corresponding figures alongside averages and ±1 SD. Statistical significance was assessed using Kruskal-Wallis ANOVA, followed by a Conover’s post-hoc test for multiple comparisons. Differences were considered statistically significant at *p* < 0.05 and are indicated in the figures with an asterisk (*). Statistical analysis was performed using OriginPro 2025b (64-bit) SR1 with plugin Post-hoc Analysis for Nonparametric Tests from OriginLab Technical Support. The figures only present statistical comparisons that directly support or reject the research hypothesis. A full statistical analysis is available in the data repository.

## Supplementary Material

Additional supporting information can be found online in the Supporting Information section.

supplementary file

## Data Availability

The data that support the findings of this study are openly available in OPARA at https://doi.org/10.25532/OPARA-1107.
