# Supplementary material for: Conductive Hydrogels for Exogenous Sensing and Cell Fate Control
Source: Adv Mater. 2026 Mar 26;38(40):e72866. doi: 10.1002/adma.72866 (PMC7619064; doi:10.1002/adma.72866)
Supplement: Supplementary file 1 — Supporting File: adma72866‐sup‐0001‐SuppMat.docx. [file ADMA-38-e72866-s001.docx]

**Supplementary Materials: Conductive Hydrogels for Exogenous Sensing and Cell Fate Control**

Teuku Fawzul Akbar^1^, Carlos Alejandro Jimenez-Rodriguez^1^, Railia Biktimirova^1^, Ilka Hermes^2^, Thomas Kurth^3^, My Duyen Pham^1^, Mikhail Tsurkan^1^, Jens Friedrichs^1^, Francis L. C. Morgan^1^, Hans Kleemann^4^, Olga Guskova^5^, Uwe Freudenberg^1^, Peter Fratzl^6^, Carsten Werner^1,7^, Christoph Tondera^1,7 + *^, Ivan R. Minev^1,8 +*^

^1^Division of Polymer Biomaterials Science, Leibniz Institute for Polymer Research Dresden, Dresden, Germany

^2^Division Physical Chemistry and Physics of Polymers, Leibniz Institute for Polymer Research Dresden, Dresden, Germany

^3^ Core Facility Electron Microscopy and Histology, Center for Molecular and Cellular Bioengineering, TUD Dresden University of Technology, Dresden, Germany

^4^Dresden Integrated Center for Applied Physics and Photonic Materials, TUD Dresden University of Technology, Dresden, Germany

^5^Division Theory of Polymers, Leibniz Institute for Polymer Research Dresden, Dresden, Germany

^6^Department of Biomaterials, Max Planck Institute of Colloids and Interfaces, Potsdam, Germany

^7^Center for Regenerative Therapies Dresden, TUD Dresden University of Technology, Dresden, Germany

^8^Else Kröner Fresenius Center for Digital Health, Medical Faculty Carl Gustav Carus, TUD Dresden University of Technology, Dresden, Germany

^+^ contributed equally

* corresponding authors [tondera@ipfdd.de](mailto:tondera@ipfdd.de), [ivan.minev@tu-dresden.de](mailto:ivan.minev@tu-dresden.de)

**Supplementary Methods**

**Analysis of maleimide-conjugated heparin**

To assess the maleimide-functionalization of heparin, samples were analyzed via ^1^H NMR, and Size-Exclusion Chromatography (SEC). ^1^H NMR experiments were performed on a Bruker Avance III (Bruker, 500 MHz) in phosphate buffered D_2_O (57 mM PO_4_^3-^, pH = 5, with DSS-*d*_6_ (Sigma-Aldrich) as an internal standard at 0.10 mM). Samples were prepared at 20 wt.% (20.0 mg/mL; NMR sample volume = 580 µL). For 1D ^1^H measurements, the integral of the maleimide protons and internal standard, alongside the known mass content, were used to determine the degree of functionalization following a described method ^1^. Over several batches (n = 4) the average number of maleimides per heparin chain was found to be 6.4 ± 1.0 (mean ± 1 SD). In addition, a 2D DOSY NMR experiment was performed to confirm the attachment of the maleimide groups to the heparin backbone. Example NMR spectra can be found in **Figure S2**. The ^1^H DOSY measurement was processed using Bruker’s Dynamic Center Software (v2.8.9).

SEC measurements were performed using an Isocratic Pump 1260 (Agilent Technologies) equipped with two PL aquagel OH-MIXED-H (8 μm) columns connected to a miniDAWN® TREOS® II light scattering detector (Wyatt Technologies), a UV detector (KNAUER Smartline 2520) and a refractive index (RI) detector (KNAUER AZURA®, RID2.1L) in an eluent composed of 0.01 M NaH_2_PO_4_ (pH = 7.0), 0.2 M NaNO_3_ and 0.02% NaN_3_ at 25 °C and at a flow rate of 1 mL/min. Samples were prepared at 3.5 mg/mL, dissolved at RT for 2 h prior to injection. The RI signal was used for apparent molecular weight and dispersity determination against PEG standards (*M*_p_ = 1450–298000 g/mol) to ensure that the maleimide-functionalization did not cause any degradation, coupling, or significant increases to dispersity. We found the maleimide functionalized heparin to have an apparent *M*_n_ of 11 500 ± 400 g/mol and *Đ* ≈ 1.55 ± 0.05. Example SEC traces can be found in **Figure S3**. We note that the values presented here are apparent (not absolute), as the highly charged Heparin chain will possess an aspect ratio that is not spherical and a PEG standard will not adequately capture the polysaccharide’s solvated behavior.

**Molecular simulations**

The Gibbs free energy of solvation ($\Delta G_{solv}$) for (pristine or functionalized) heparin was determined using thermodynamic integration (TI), implemented in Materials Studio 7.0 (BIOVIA, Dassault Systèmes, Materials Studio 7.0, 2014) with the COMPASS force field ^2^. All calculations were carried out under isothermal-isobaric conditions at 298 K and 1 atm. The TI protocol models the progressive coupling of a solute with its solvent environment via a coupling parameter $\lambda\in[0, 1]$, where $\lambda$ = 0 corresponds to a non-interacting solute and $\lambda$ = 1 to a fully interacting system.

The total solvation free energy was obtained by numerically integrating the ensemble average of the derivative of the Hamiltonian ($H$) with respect to λ:

$\Delta G_{solv}=\int_{0}^{1} \left\langle\frac{\delta H\left( \lambda\right)}{\delta\lambda} \right\rangle d\lambda$ Equation 1.

The calculation was partitioned into three distinct stages. In the first ($\Delta G_{1}$), partial charges were gradually removed from the solute in vacuum, keeping van der Waals (vdW) interactions intact. In the second ($\Delta G_{2}$), an uncharged solute was solvated by incrementally enabling vdW interactions. In the final step ($\Delta G_{3}$), the electrostatic interactions were restored in the solvated environment. Each thermodynamic leg was sampled using 20 $\lambda$ windows, with 1 ns of molecular dynamics performed per window. Starting configurations were extracted from prior full-atomistic MD simulations (**Figure S4**). The resulting $\Delta G_{solv}$ is a sum of these three contributions.

The classical Hildebrand solubility parameter (δ), defined as the square root of the cohesive energy density, is typically derived from the enthalpy of vaporization. However, this approach is inapplicable to charged or thermally labile systems such as heparin, which cannot be vaporized without degradation. To circumvent this limitation, we estimate δ from the free energy of hydration $\Delta G_{solv}$. When normalized by molar volume, this metric provides a physically grounded proxy for cohesive energy density. This substitution is particularly justified for polyelectrolytes like native heparin and its maleimide-functionalized analogues, where hydration is governed by a complex interplay of electrostatics, hydrogen bonding, and dispersion.

**Rheological characterization of sGAGh and PEDOT:sGAGh**

Rheological characterization was performed on an Anton Paar MCR301 equipped with a solvent trap to prevent hydrogel drying, using a 25 mm parallel plate geometry. Hydrogels were prepared according to the procedure described in the main text, but with a diameter of 25 mm and a thickness of 640–770 µm. Immediately before loading into the rheometer, hydrogel samples were carefully dabbed dry using KimTech^TM^ wipes to wick away residual surface moisture and limit slippage. Loading was achieved using a closing profile setting an applied normal force of 1 N to ensure good contact. Frequency sweeps were performed from 100–1 rad/s at 1% strain, followed immediately by strain sweeps from 0.1–100% strain at 1 Hz for each sample. Total measurement time was 20 min.

**MMP-Cleavable Peptide Synthesis**

*Solid-Phase Synthesis of MMP.* The peptide Ac-GC(StBu)GGPQGIWGQGGCG-NH₂ (MMP) was synthesized via Fmoc-based solid-phase peptide synthesis on a Liberty Blue™ HT12 microwave peptide synthesizer (CEM Corporation) using a 30 mL reaction vessel at a 0.25 mmol scale (four parallel 0.25 mmol batches). Resin loading and reagent quantities were calculated using the integrated Liberty Blue software. Fmoc-Rink amide AM resin (loading 0.40–0.75 mmol/g, 100–200 mesh; Iris Biotech) was employed, affording a *C*-terminal amide (CONH₂) upon cleavage. The resin was swollen in 10 mL of 50:50 DMF/DCM (*v*/*v*) prior to synthesis. Dimethylformamide (DMF; Iris Biotech Sol-004.9025) and dichloromethane (DCM; Iris Biotech) were used as solvents. Fmoc-protected amino acids from Iris Biotech were prepared as 0.4 M solutions in DMF using the following: Fmoc-L-Cys(StBu)-OH (FAA1575), Fmoc-L-Cys(Trt)-OH (FAA1040), Fmoc-L-Gln(Trt)-OH (FAA1043), Fmoc-L-Gly-OH (FAA1050), Fmoc-L-Ile-OH (FAA1110), Fmoc-L-Pro-OH*H2O (FAA1225), and Fmoc-L-Trp(Boc)-OH (FAA1185). Coupling reactions were mediated by *N*,*N*'-Diisopropylcarbodiimide (0.5 M in DMF; Iris Biotech RL-1015.0500) and ethyl cyano(hydroxyimino)acetate (Oxyma Pure, 1.0 M in DMF; Iris Biotech RL-1180-0100). Fmoc deprotection was achieved using 20% (*v*/*v*) piperidine (Iris Biotech Sol-010.2500) in DMF. The synthesis followed the standard 0.25 mmol microwave-assisted coupling and deprotection cycles (C-terminus: amide; resin type: Rink; standard resin swelling; final deprotection at 75 °C). The *N*-terminus was acetylated using 10% acetic anhydride (>99%, Alfa Aesar) in DMF. Upon completion of the automated synthesis, the resin was washed with DMF using a Duran® glass filter (POR 2, 50–75 mL; DWK Life Sciences) and dried under nitrogen flow.

*Peptide Cleavage and Isolation.* Cleavage of the combined parallel syntheses was achieved using a cocktail composed of trifluoracetic acid (TFA, Iris Biotech SOL-011.500; 85 mL), phenol (Carl Roth 0040.1; 5.0 g), triisopropylsilane (Iris Biotech RL-1102.0050; 0.625 mL), Milli-Q^TM^ (5.0 mL), and DCM (2.5 mL). The reaction mixture was magnetically stirred in the cleavage cocktail at room temperature (RT) for 4 h. If required, 0.5–1 mL DCM was added to ensure a homogenous resin suspension. The resin was removed by filtration (Duran® POR 2 glass filter) and washed with additional cleavage solution. The filtrate containing the crude peptide was precipitated into diethyl ether (Iris Biotech SOL-005.2500) and left overnight at 4 °C. The precipitated peptide was filtered (Duran® POR 4 glass filter) and sequentially washed with diethyl ether (3x), DCM, then diethyl ether (3x). Between each wash, the peptide was dried under nitrogen flow.

*Preparative HPLC Purification.* Purification of the crude peptide was performed by preparative reversed-phase HPLC (Infinity II 1260 System, Agilent Technologies) using a Luna® 5 µm C18(2), 100 Å column (250 × 30 mm, AXIA packed; Phenomenex). The flow rate was set to 20.0 mL/min. Eluent A consisted of 5% ACN in Milli-Q^TM^, containing 0.1% TFA. Eluent B consisted of 0.1% TFA in ACN. For purification, 360 mg crude peptide were dissolved in 15 mL of 50:50 ACN/Milli-Q^TM^ in a 50 mL centrifuge tube then diluted with Milli-Q^TM^ to a total volume of 45 mL. Prior to injection, the solution was filtered using a Rotilabo® CME syringe filter (0.8 µm, 25 mm; Roth 499868413). For each run, 15 mL of the filtered solution were injected. Elution was monitored by DAD detection, and the main product fraction was manually collected via the instrument control software. To recover residual product retained on the column, two additional runs using 20 mL Milli-Q^TM^ were carried out, and the relevant fractions were combined. To neutralize residual TFA from the mobile phase, 13.06 µL of 1.0 M HCl were added per 10 mL of collected fraction (corresponding to 0.01306 mmol, i.e., one tenth of the molar amount of TFA present in 10 mL of eluate). Acetonitrile was removed under reduced pressure, and the remaining aqueous solution was freeze-dried to afford the purified peptide as a white, fluffy solid. Typical peptide yields were ≈65%

*HPLC-MS Analysis.* Analytical HPLC-MS measurements were carried out on an Agilent Infinity 1260 II LC system equipped with a binary pump and coupled to a 6230B Time-of-Flight (TOF) mass spectrometer (Agilent Technologies). UV-Vis detection was achieved using a diode array detector (DAD G7115A, Agilent Technologies) equipped with a 1 cm path length flow cell. Chromatographic separation was performed on a Luna® 5 µm C18(2) column (250 × 3 mm, 100 Å; Phenomenex, Torrance 00G-4252-Y0) maintained at 25 °C. The flow rate was set to 0.500 mL/min. The total runtime per sample was 40 min. Gradient elution was applied as follows: 0.00 min, 100% solvent A; 30.00 min, 40% A/60% B; 32.00 min, 0% A/100% B; 34.00 min, 100% A, followed by re-equilibration. Solvent A consisted of 95% acetonitrile (ACN, LC-MS grade, LiChrosolv®, Merck 1.00029.2500), 5% Milli-Q^TM^, and 0.1% formic acid (AnalaR NORMAPUR®, VWR 20318.297). Solvent B consisted of 5% ACN in Milli-Q^TM^ containing 0.1% formic acid. The peptide sample was dissolved in 50:50 ACN /Milli-Q^TM^ and 5 µL were injected. Detection with the DAD was monitored at 210.4 nm. Mass spectrometric analysis employed dual electrospray ionization (ESI) in positive ion mode, with nitrogen serving as both the nebulizing and desolvation gas. HPLC-MS data are shown in **(Figure S18)** for both the synthesized, and an identical purchased peptide.

*Preparation of PEG-MMP.* The Ac-GC(StBu)GGPQGIWGQGGCG-NH₂ peptide (synthesized or purchased) was subsequently conjugated to a 4-arm PEG-maleimide (10K, Jenkem) using standard EDC/NHS coupling, followed by deprotection of the cysteine-StBU using a 5-fold molar excess Tris(2-carboxyethyl)phosphine hydrochloride (TCEP, ≈3.5 mM; Iris Biotech LS-3405) adjusted to pH 7–8 using NaOH.

**Supplementary Table**

| **M_6_ sGAGh** | | **Concentration (nmol/µL)** | | |
| --- | --- | --- | --- | --- |
| **P (µmol/mL)** | **M** | **Heparin - maleimide** | **PEG - maleimide** | **PEG - SH** |
| 0 | 6 | 0 | 1.68 | 1.68 |
| 2 | 6 | 0.03 | 1.66 | 1.68 |
| 10 | 6 | 0.15 | 1.53 | 1.68 |
| 140 | 6 | 2.11 | 0 | 2.11 |
| **M_0_ sGAGh** | | **Concentration (nmol/µL)** | | |
| **P (µmol/mL)** | **M** | **Heparin** | **PEG - COOH** | **PEG - NH2** |
| 10 | 0 | 0.51 | 5.14 | 5.65 |
| 130 | 0 | 5.65 | 0 | 5.65 |

**Supplementary Table 1: The composition of different sGAGh template hydrogels is shown.**

**Supplementary Figures**

**Figure S1: Oxidative polymerization of PEDOT.**

Schematic illustration of the oxidative chemical polymerization of 3,4-ethylenedioxythiophene (EDOT) to form poly(3,4-ethylenedioxythiophene) (PEDOT) using ammonium persulfate (APS) as the oxidant. APS oxidizes EDOT to generate two radical cations, which subsequently couple to produce an EDOT dimer. This coupling is followed by deprotonation. The process repeats to add more EDOT monomers to the polymer chain. The oxidative polymerization process introduces charge carriers in the form of polarons and bipolarons on the PEDOT backbone, which are stabilized by counter-ions (not shown).


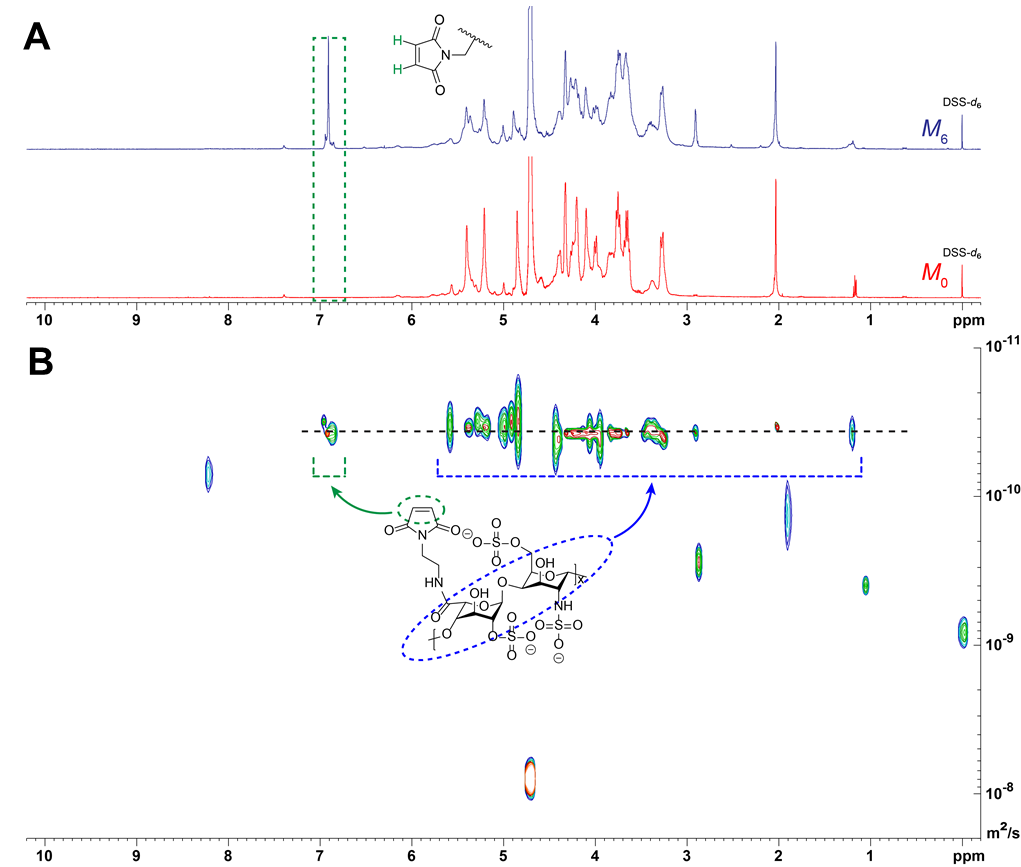


**Figure S2: Example NMR spectra of heparin and maleimide-functionalized heparin.**

**(A)** Comparing the ^1^H NMR spectrum (500 MHz, D_2_O with 57 mM PO_4_^3-^, pH = 5, and DSS-*d*_6_ as an internal standard at 0.10 mM) of the pure heparin to the spectrum of *M*_6_ heparin-maleimide shows the vinylic proton signals from the maleimide at 6.9 ppm. These were integrated relative to the DSS-*d*_6_ internal standard signal at 0 ppm to determine their concentration and consequently the degree of functionalization. **(B)** To demonstrate that the maleimide group is attached to the heparin backbone, a ^1^H DOSY was also run under identical conditions. Both the maleimide signal and the signals from the backbone of the heparin chain yield the same diffusion coefficient as indicated by the black dotted line. The DSS-*d*_6_, residual DHO, and some trace impurities also give rise to DOSY signals at higher diffusion rates.

**Figure S3: Size exclusion chromatography (SEC) analysis of maleimide-functionalized Heparin.**

The maleimide functionalization of heparin shows an increase in apparent *M*_n_. This increase is unsurprising as the presence of maleimide is expected to slightly reduce chain flexibility and increase the hydrodynamic radius of the functionalized polysaccharide. The peak at 19.5 min is from the running buffer (0.01 M NaH_2_PO_4_ (pH=7.0) + 0.2 M NaNO_3_ + 0.02 % NaN_3_).


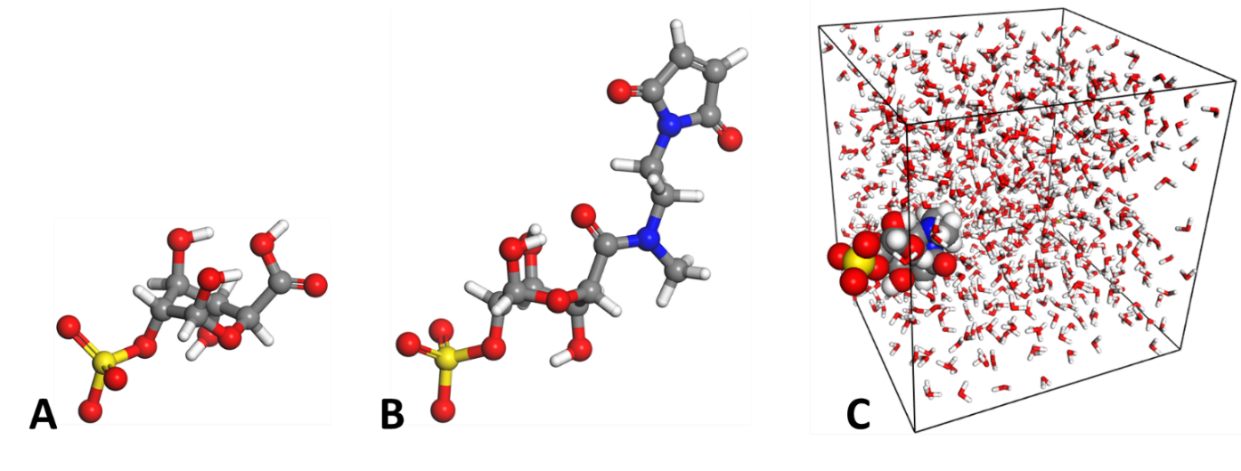


**Figure S4: Molecular Dynamics calculations with non-modified and maleimide modified Heparin.**

A representative saccharide unit of **(A)** non-modified and **(B)** maleimide-modified heparin. **(C)** A solute molecule (here with maleimide) dissolved in water in a cubic simulation box for the calculation of the Gibbs free energy of hydration $\Delta G_{solv}$ . Values for $\Delta G_{solv}$ were significantly more negative for unmodified heparin (-46.48 ± 0.74 kcal/mol) compared to maleimide-modified heparin (-32.21 ± 0.64 kcal/mol), indicating a reduced water affinity upon substitution. To gain further insight, we calculate the Hildebrand solubility parameter (δ) for unmodified monomeric unit and unit bearing maleimide. The δ values markedly decreased from 40.4 mPa^1/2^ for the carboxylated unit to 26.5 mPa^1/2^ upon maleimide substitution, reflecting reduced polarity and water affinity.

Initial molecular structures were constructed using Materials Studio and subjected to geometry optimization via molecular mechanics. Each solute molecule, modeled as a molecular ion paired with a sodium counterion, was placed in a cubic simulation box containing 700 water molecules. The initial solution density was set to 0.7 g/cm³. To eliminate steric clashes between the solute, counterion, and solvent molecules, the system was equilibrated prior to production runs. Subsequently, NPT ensemble simulations were performed at 298 K (Nose thermostat) and 10^-4^ GPa (Berendsen barostat) to achieve realistic water density. The final equilibrated density reached 0.98 g/cm³. Each simulation was run for 10 ns for both solutes. A representative snapshot from the end of the trajectory was used as the starting configuration for thermodynamic integration. The Hildebrand solubility parameter (δ) is defined as the square root of the cohesive energy density, which represents the internal energy required to vaporize a substance per unit volume: $\delta=\sqrt{\frac{E_{coh}}{V_{m}}}=\sqrt{\frac{\left| \Delta G_{solv} \right|}{V_{m}}}$. The molecular volumes are calculated using the Connolly surface method, which defines the volume enclosed by the solvent-accessible surface traced by a water-sized probe sphere with a radius of 1.4 Å rolling over the solute molecule.

|  |
| --- |
| 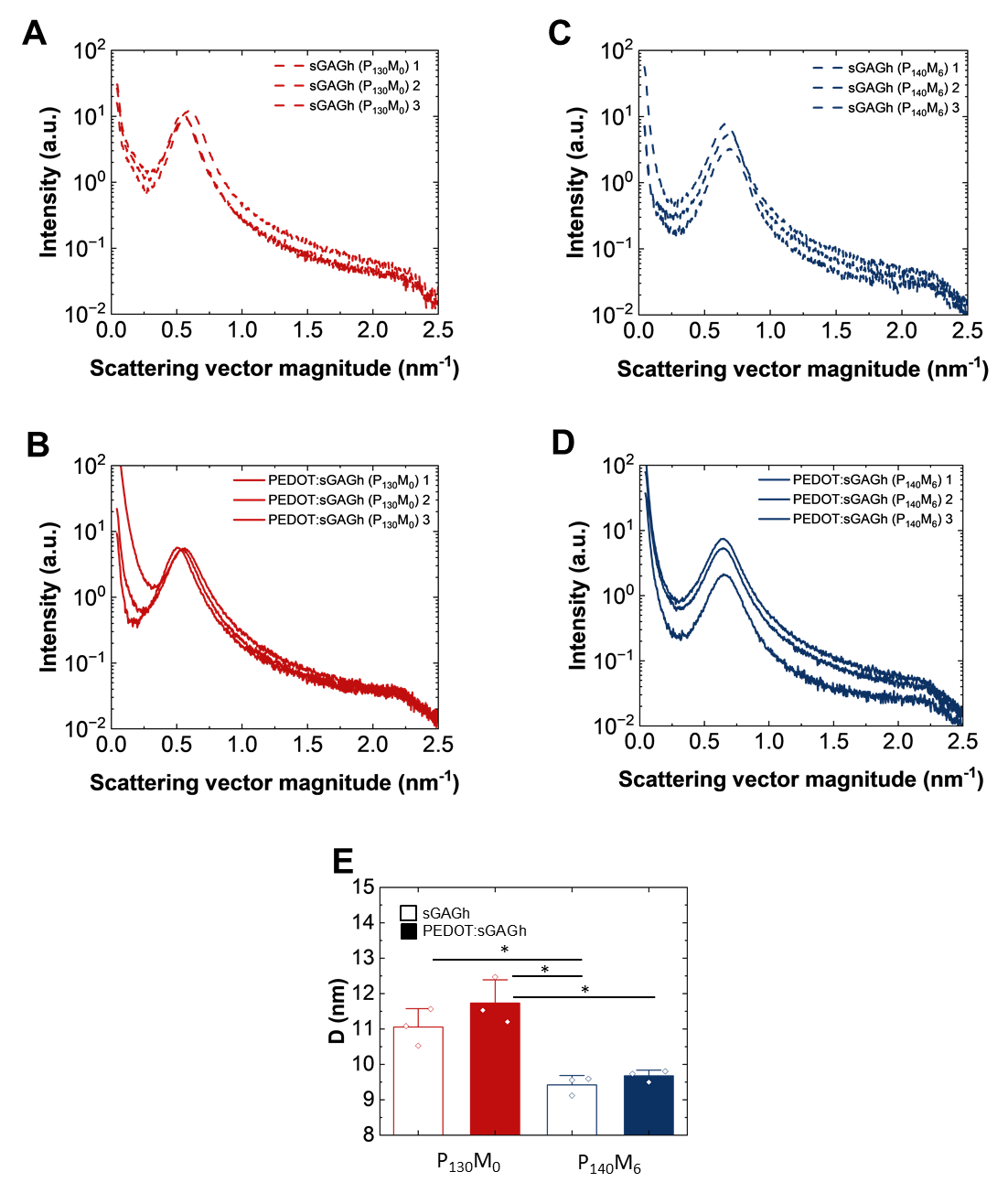 |
| **Figure S5: Small Angle X-ray scattering analysis of sGAGh and PEDOT:sGAGh.**  Radially integrated diffraction pattern intensities from three distinct locations within a representative sample of **(A)** sGAGh (P_130_M_0_), **(B)** PEDOT:sGAGh (P_130_M_0_), **(C)** sGAGh (P_140_M_6_) and **(D)** PEDOT:sGAGh (P_140_M_6_). Samples were prepared according to the same procedure as for TEM, but without uranyl acetate staining. **(E)** D-period calculated from spectra in (A-D). In real space, the D-period is calculated according to $D=\frac{2\pi}{q_{1}}$ where $q_{1}$is the location of the diffraction peak.  Data from all samples show a pronounced peak which is likely the signature of a semicrystalline structure. The analysis then shows a similar D-period for all materials ranging from approximately 9nm to 13nm. The D-period is indicative of the space between crystalline regions and thus the size of void structures in the materials ^3^. The size of voids is likely underestimated as the network expands when hydrogels are swollen with PBS. The largest protein in our study (VEGF, 38.2 kDa) is estimated to have a hydrodynamic radius of 5nm ^4^, which is significantly smaller than the estimated void sizes in both sGAGh and PEDOT:sGAGh materials. This indicates that the bulk of the hydrogels is accessible to macromolecular cargo. Bar plot (E) shows individual data points, averages and + 1 S.D. Statistical significance was assessed using Kruskal-Wallis ANOVA, followed by a Conover’s post-hoc test for multiple comparisons. Differences were considered statistically significant at p < 0.05 and are indicated with *. |

**
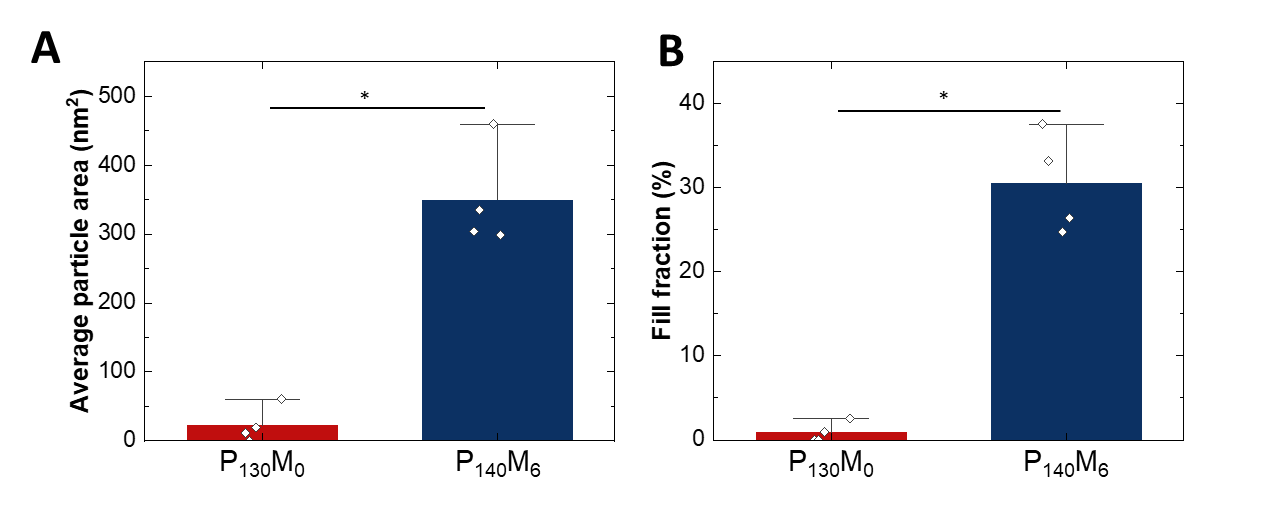
**

**Figure S6: Quantitative analysis of conductive AFM measurements.**

**(A)** Quantification of the conductive particle area for particles larger than 50 nm². **(B)** Fill fraction of the conductive particles as measured by cAFM, for the different PEDOT:sGAGh materials (P_130_M_0_ and P_140_M_6_). The quantification supports the hypothesis of a percolating conductive network in the PEDOT:sGAGh material P_140_M_6_. Bar plots (A and B) show individual data points, averages and + 1 S.D. Statistical significance was assessed using Mann-Whitney test. Differences were considered statistically significant at p < 0.05 and are indicated with *.


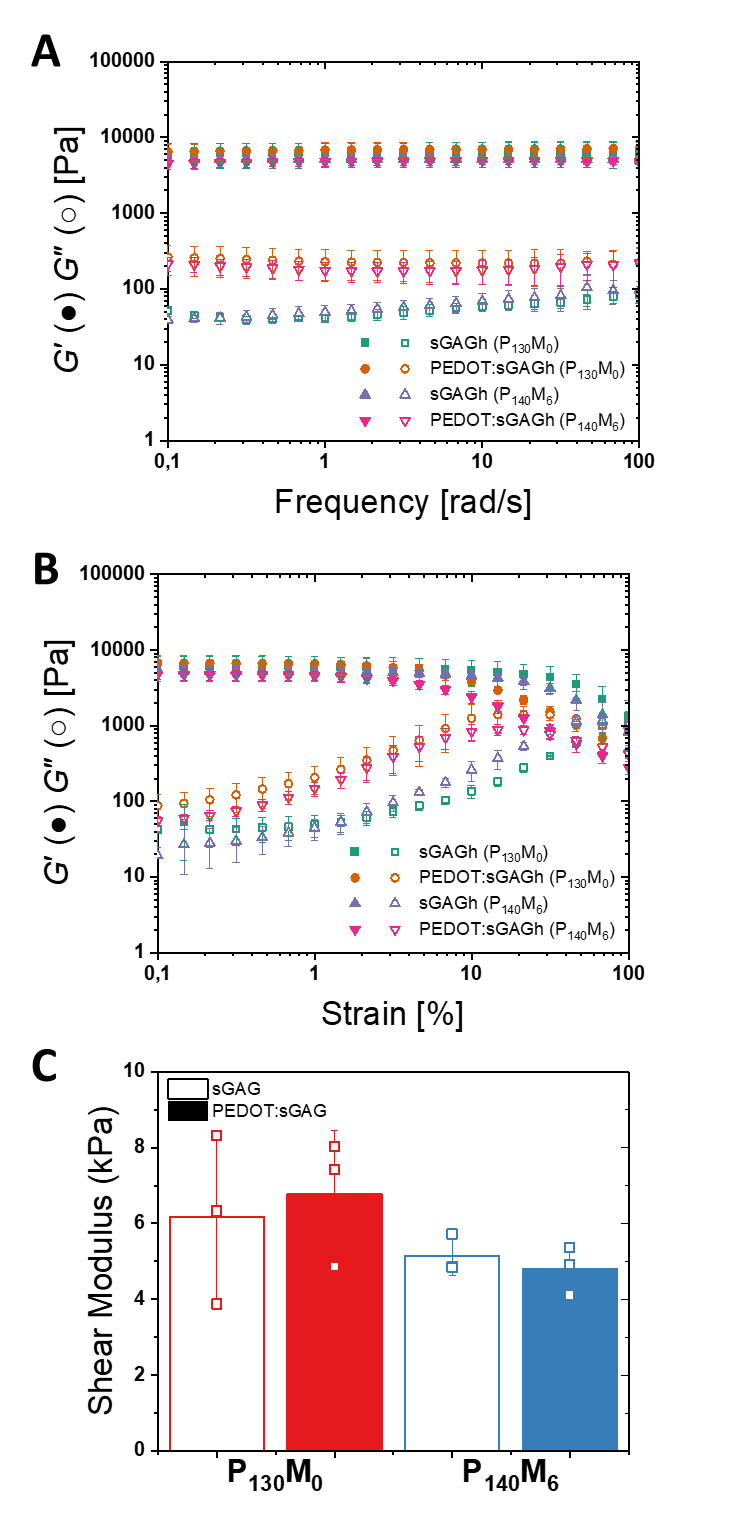


**Figure S7: Rheological characterization of sGAGh and PEDOT:sGAGh.**

**(A)** Frequency sweeps (100–0.1 rad/s @ 1% strain) of both sGAGh and PEDOT:sGAGh reveal elastic and frequency independent behavior of the storage (*G*′; closed symbols) and loss (*G*″; open symbols) modulus. The PEDOT:sGAGh exhibit a slightly higher loss modulus. **(B)** strain sweeps (0.1–100 % strain @ 1 Hz) show a consistent shear modulus with slightly increasing loss modulus up to ≈2% strain. We note that some slippage was observed at higher (beyond ≈10%) strains. **(C)** The shear modulus values were taken from the frequency sweep data at 1 rad/s. Bar plot shows individual data points, averages and ± 1 S.D. No statistical significance was detected using Kruskal-Wallis ANOVA.

**
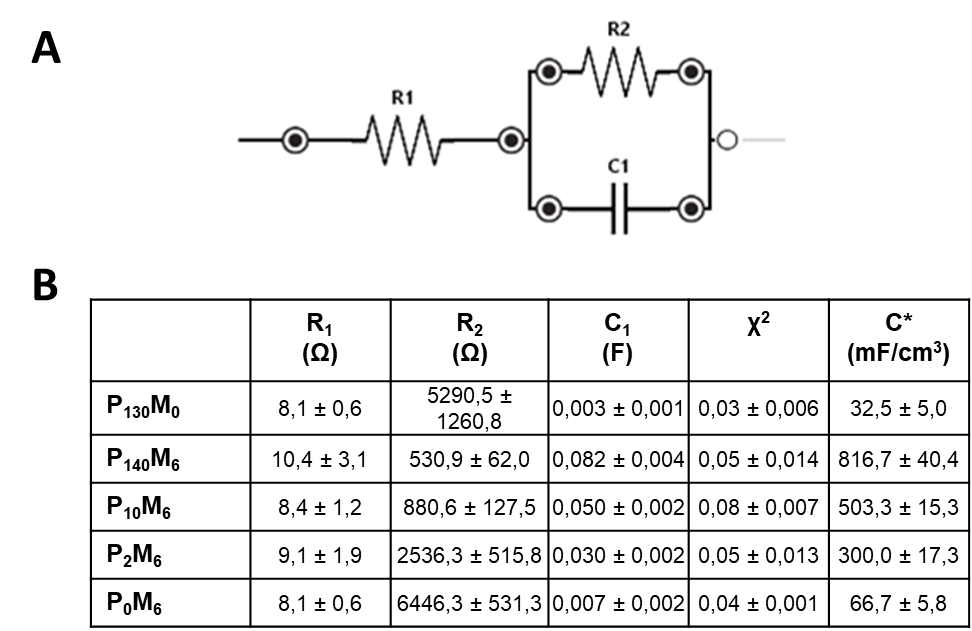
**

**Figure S8: Equivalent circuit fitting.**

**(A)** Electrochemical impedance spectra were fitted to the Randles equivalent circuit, which includes a resistor R_1_ (electrolyte resistance), connected to a parallel combination of a resistor R_2_ (Ohmic resistance of the PEDOT network) and a capacitor C_1_ (volumetric capacitance of PEDOT:sGAGh). **(B)** Fitting results for R_1_, R_2_ and C_1_ and the volume normalized capacitance (C*) for different PEDOT:sGAGh formulations. Low χ²-values indicate a good fit between measured and simulated spectra.


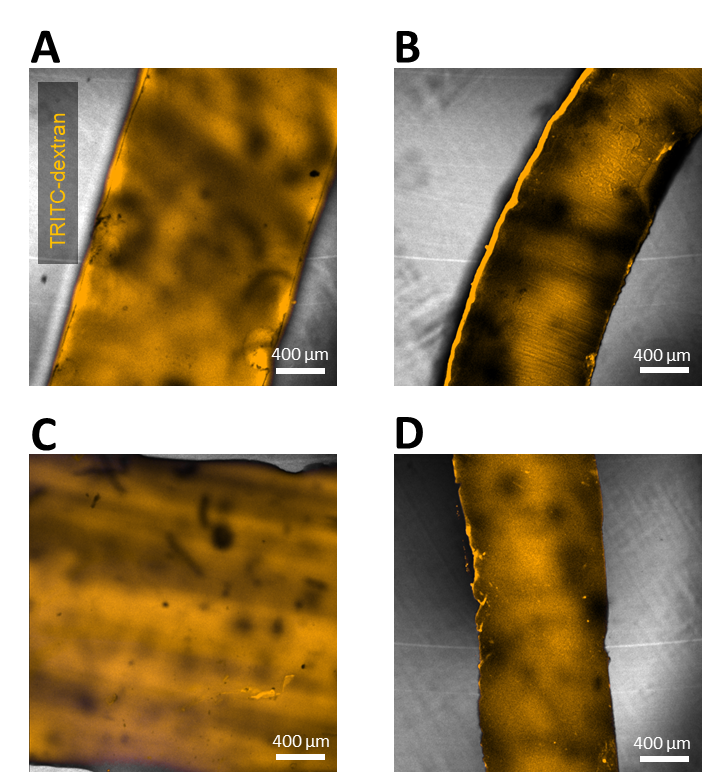


**Figure S9: Perfusion of PEDOT:sGAGh with a model macromolecule.**

Disks of PEDOT:sGAGh with a diameter of 11 mm and a volume of 100 µL are synthesized. Disks are then incubated in a 200 µg/mL solution of TRITC labelled dextran (20 kDa, TdB labs) in PBS overnight. After incubation, 800 µm slices are cut using a vibratome and imaged with a confocal light microscope (Andor Dragonfly). **(A)** – P_10_M_0_, **(B)** – P_130_M_0_, **(C)** – P_10_M_6_, **(D)** – P_140_M_6_). Images indicate that the bulk of the hydrogel is accessible to macromolecular cargo (here dextran).


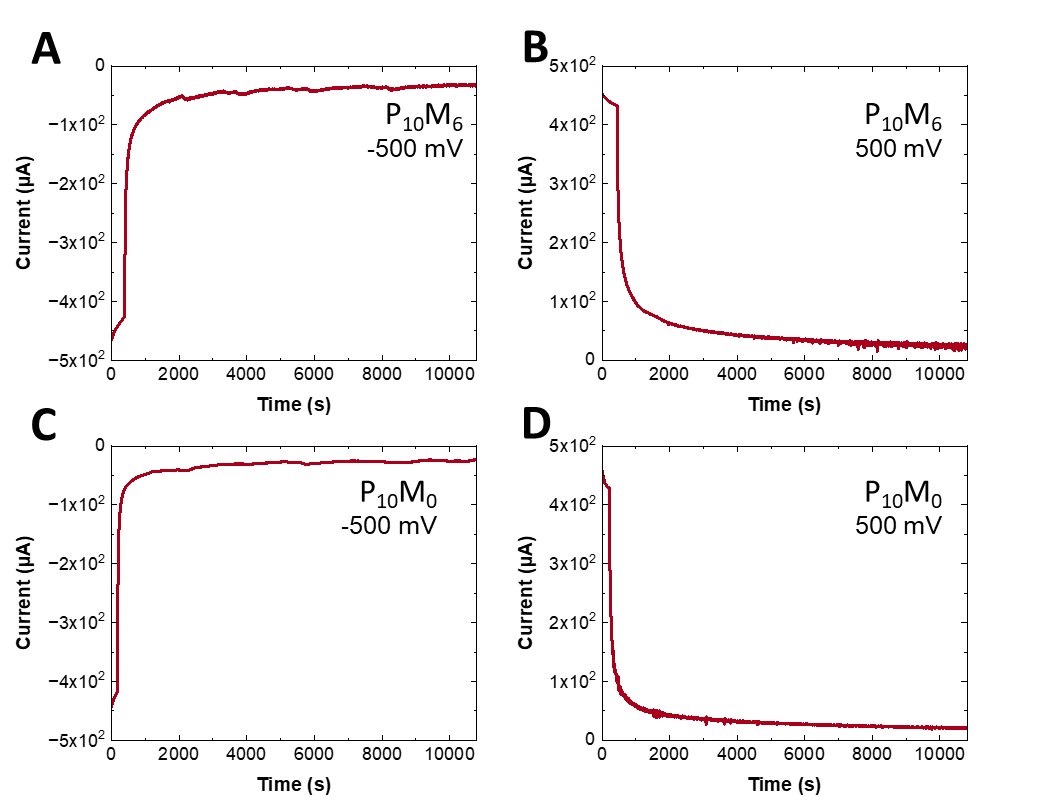


**Figure S10: Chronoamperometry of PEDOT:sGAGh integrated on gold meshes.**

Chronoamperometry measurements obtained during electrically triggered drug release or retention from P_10_M_6_ **(A, B)** and P_10_M_0_ **(C, D)** PEDOT:sGAGh materials. A constant potential of -500 mV **(A, C)** or +500 mV **(B, D)** vs. Ag/AgCl was applied for 3 hours in PBS.


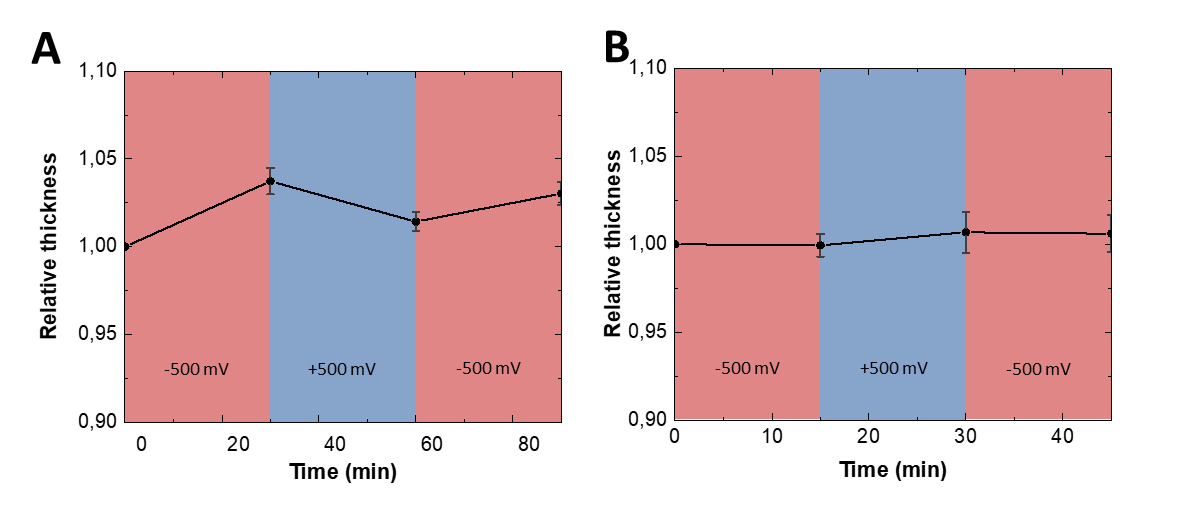


**Figure S11: Quantification of electrochemically induced PEDOT:sGAGh swelling.**

PEDOT:sGAGh samples with 0.5 mm nominal thickness before any swelling is fabricated on gold-coated polyimide film. This construct is taped onto a glass slide and is positioned vertically inside a 6-well cell culture plate filled with PBS. This setup allows observation of hydrogel thickness using a confocal microscope (Andor Dragonfly). We used PEDOT:sGAGh as working electrode and alternately applied -500 mV or +500 mV (vs. Ag/AgCl wire) via a potentiostat (Autolab PGSTAT204) to observe any changes in thickness. **(A)** Sample P_130_M_0_ exhibited thickness change of less than 5% at –500 mV, followed by contraction back to the initial thickness upon potential reversal. **(B)** Sample P_10_M_6_ showed no significant (Kruskal-Wallis ANOVA) thickness change in response to the applied potentials. The PEDOT:sGAGh variants used for protein experiments had the same anionic space charge (P = 10 µmol/mL) as in (B). Data points show averages (n = 4) and ± 1 S.D.


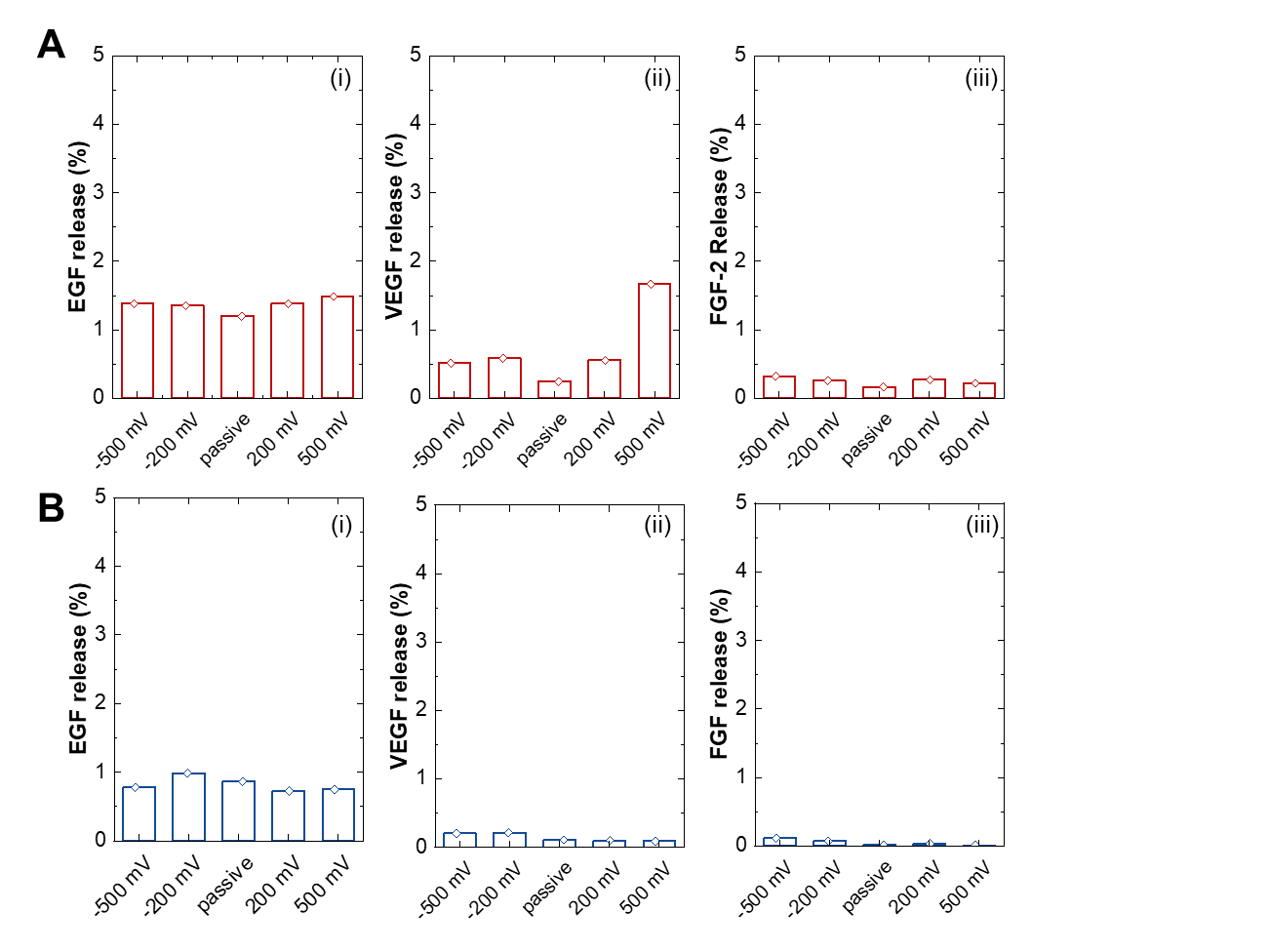


**Figure S12: Electrically stimulated growth factor release from sGAGh (without PEDOT).**

sGAGh samples (n = 1 per condition) were formed on gold meshes and loaded with the cocktail of growth factors using the same protocol as described for PEDOT:sGAGh materials. The fraction of initially loaded protein released under electrical stimulation (8 h, constant potential) and passive conditions (8 h, open circuit) for **(A)** P_10_M_0_ and **(B)** P_10_M_6_ formulations of sGAGh respectively are shown. Panels indicate (i) EFG, (ii) VEGF, and (iii) FGF-2.


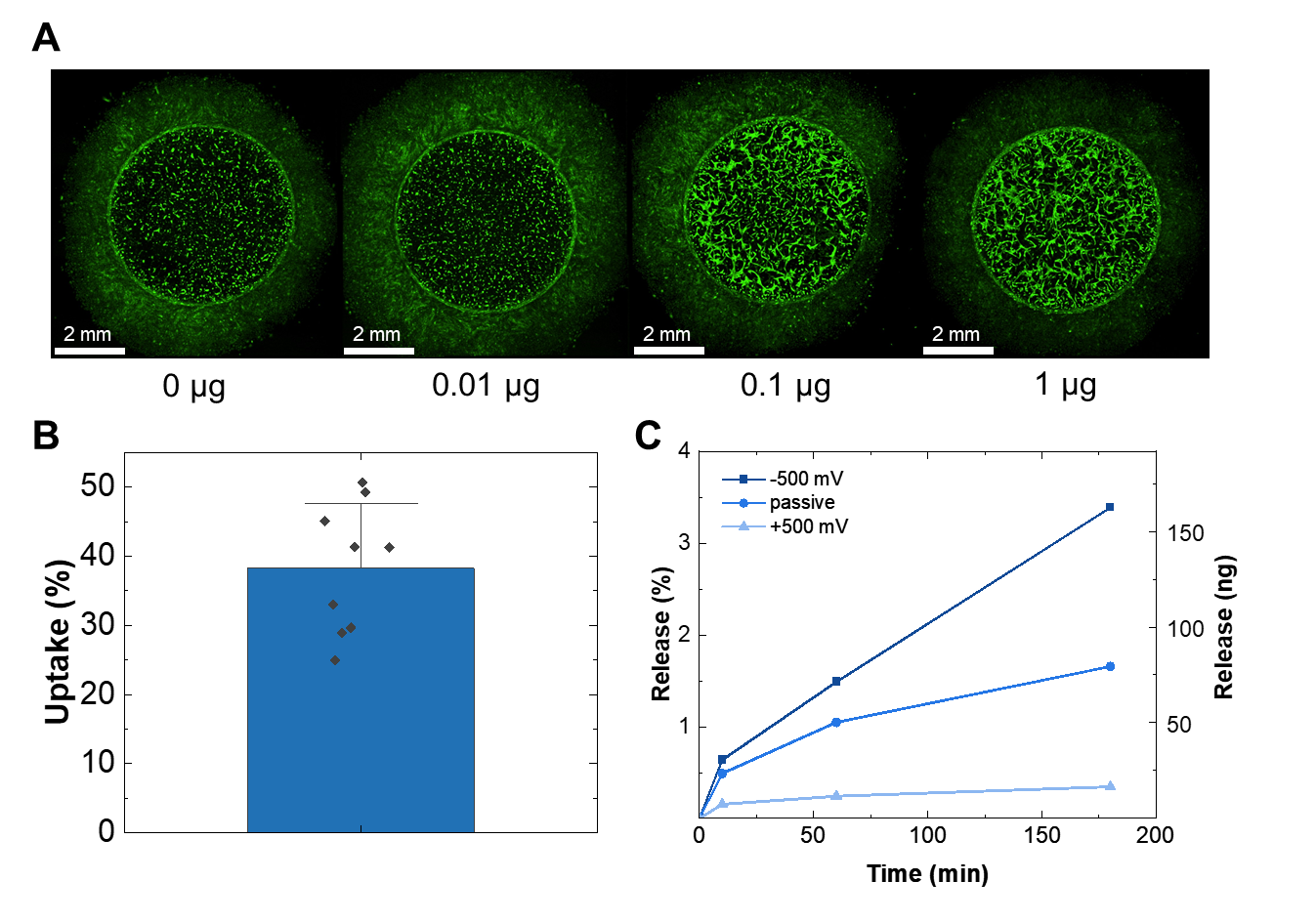


**Figure S13: Determination of VEGF loading for HUVEC differentiation experiments.**

**(A)** To determine the dose required to stimulate a response, HUVEC cells embedded in MMP-modified sGAGh (15 µL hydrogel droplets) are exposed to varying amounts of supplemental VEGF. Here we assume that nearly all of the supplemental VEGF is sequestered inside the sGAGh and available to the embedded cells. We determined 100 ng as the minimum effective (threshold) dose needed to stimulate a response in HUVECs in this system. **(B)** The fraction of VEGF taken up (sequestered) by PEDOT:sGAGh (P_10_M_0_). Bar graph shows individual data points, average and + 1 S.D. **(C)** Considering the threshold dose determined in (A) and the uptake fraction in (B), PEDOT:sGAGh were loaded with 4 µg/mL VEGF in 2.5 mL of PBS + 0.1% BSA. Dynamic release curve from PEDOT:sGAGh verified that the amount of VEGF passively released over 3 hours is close to the threshold needed to stimulate HUVEC cells. Applying reducing or oxidizing potentials then allows us to establish approximately 10-fold difference in the amount of VEGF available to cells embedded in sGAGh. The total volume of sGAGh hosting the cells is kept the same throughout all experiments. Under the assumption that sGAGh sequesters nearly all VEGF from the media, this ensures cells are exposed to a traceable amount of growth factor. n = 1 per condition.


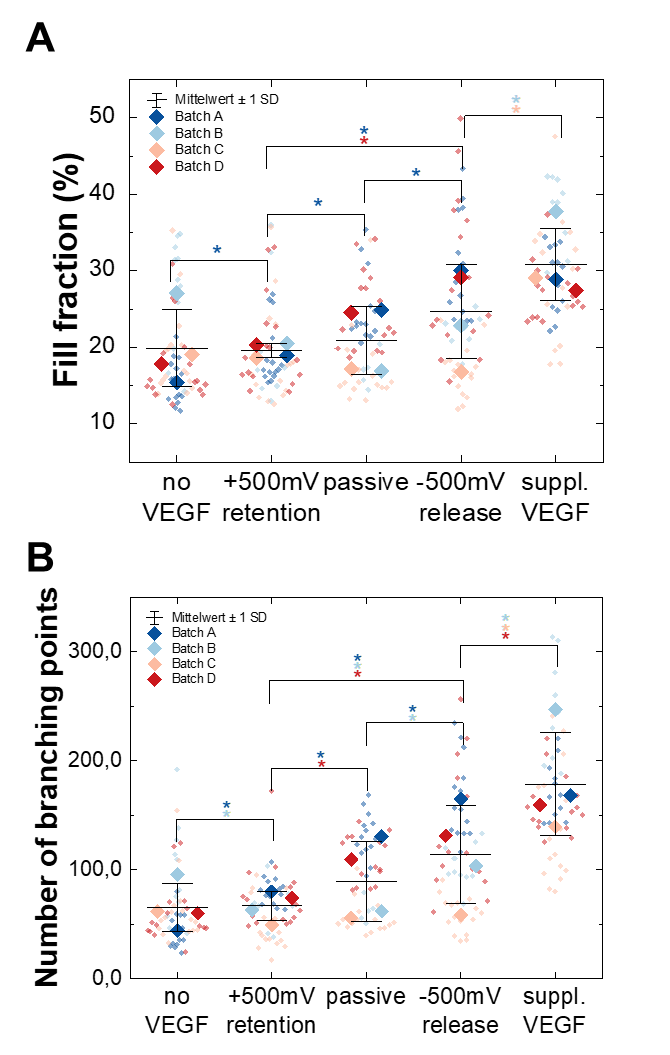


**Figure S14: Supplementary analysis of HUVECs responding to electrically controlled VEGF release.**

**(A)** Vessel fill fraction in % and **(B)** number of branching points formed by HUVECs. Measurements were taken from individual fields of view (small symbols) within the internal volume of sGAGh hydrogels. The experiment is repeated four times (Batch A–D). Large symbols indicate batch averages, horizontal lines indicate grand averages and whiskers indicate ± 1 S.D. Statistical significance was assessed using Kruskal-Wallis ANOVA, followed by a Conover’s post-hoc test for multiple comparisons. Differences were considered statistically significant at p < 0.05 and are indicated with *.


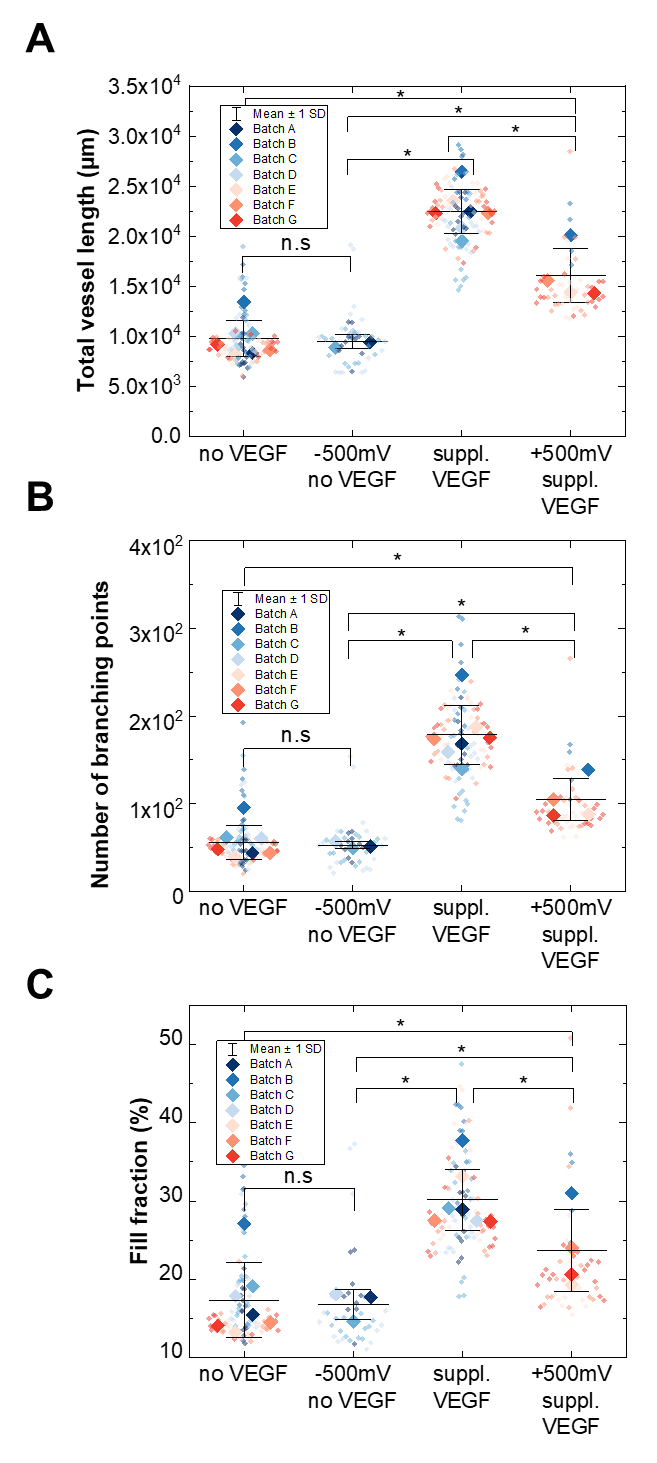


**Figure S15: Decoupling the effects of electrical stimulation and growth factor release.**

Additional controls were preformed to decouple the effects of electrical stimulation and VEGF release. Application of reducing potentials (-500 mV, no VEGF) to PEDOT:sGAGh that was not loaded with growth factor did not produce HUVEC differentiation. At the same time, differentiation was rescued by supplemental VEGF added to cultures stimulated by application of oxidative potential (+500 mV, supplemental VEGF) to PEDOT:sGAGh that was not loaded with growth factor. Quantification of **(A)** total vessel length, **(B)** density of branching points, and **(C)** vessel fill fraction % from the various control experiments. Measurements were taken from individual fields of view (1240µm x 1240 µm, small symbols) within the internal volume of sGAGh hydrogels. The experiments were repeated across at least three independent batches (A–G), horizontal lines indicate grand averages and whiskers indicate ± 1 S.D. For statistical analysis, data points from the batches were pooled together. Statistical significance was assessed using Kruskal-Wallis ANOVA, followed by a Conover’s post-hoc test for multiple comparisons. Differences were considered statistically significant at p < 0.05 and are indicated with *.


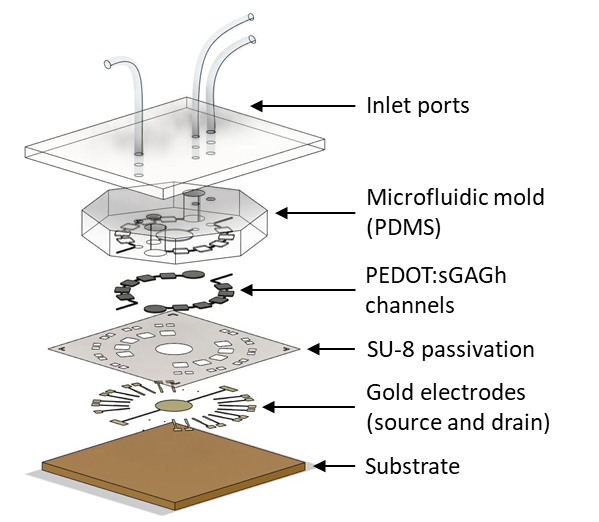


**Figure S16: Incorporation of PEDOT:sGAGh as channel material in OECTs.**

Exploded view of the microfluidic molding process for PEDOT:sGAGh channels. Substrates (polyimide or glass) were coated with a chromium/gold (3/60 nm) layer and metal was photolithographically structured to form source and drain electrodes. They were subsequently passivated with a 4 µm SU-8 layer. Separately, PDMS molds were derived from SU8-2050 positive structures on silicon. The required antiadhesive character of the soft lithography mold was achieved by functionalization with the hydrophobic silane tridecafluoro-1,1,2,2-tetrahydrooctyl. The molds included reservoirs and inlet/outlet ports to allow sGAGh hydrogel precursor perfusion by vacuum filling. The layers were aligned and clamped between two glass slides; the upper slide was equipped with inlet ports connected to the PDMS mold to facilitate perfusion of the sGAGh precursor solution. Following mold removal, devices were subjected to oxidative polymerization of PEDOT to form PEDOT:sGAGh channels.


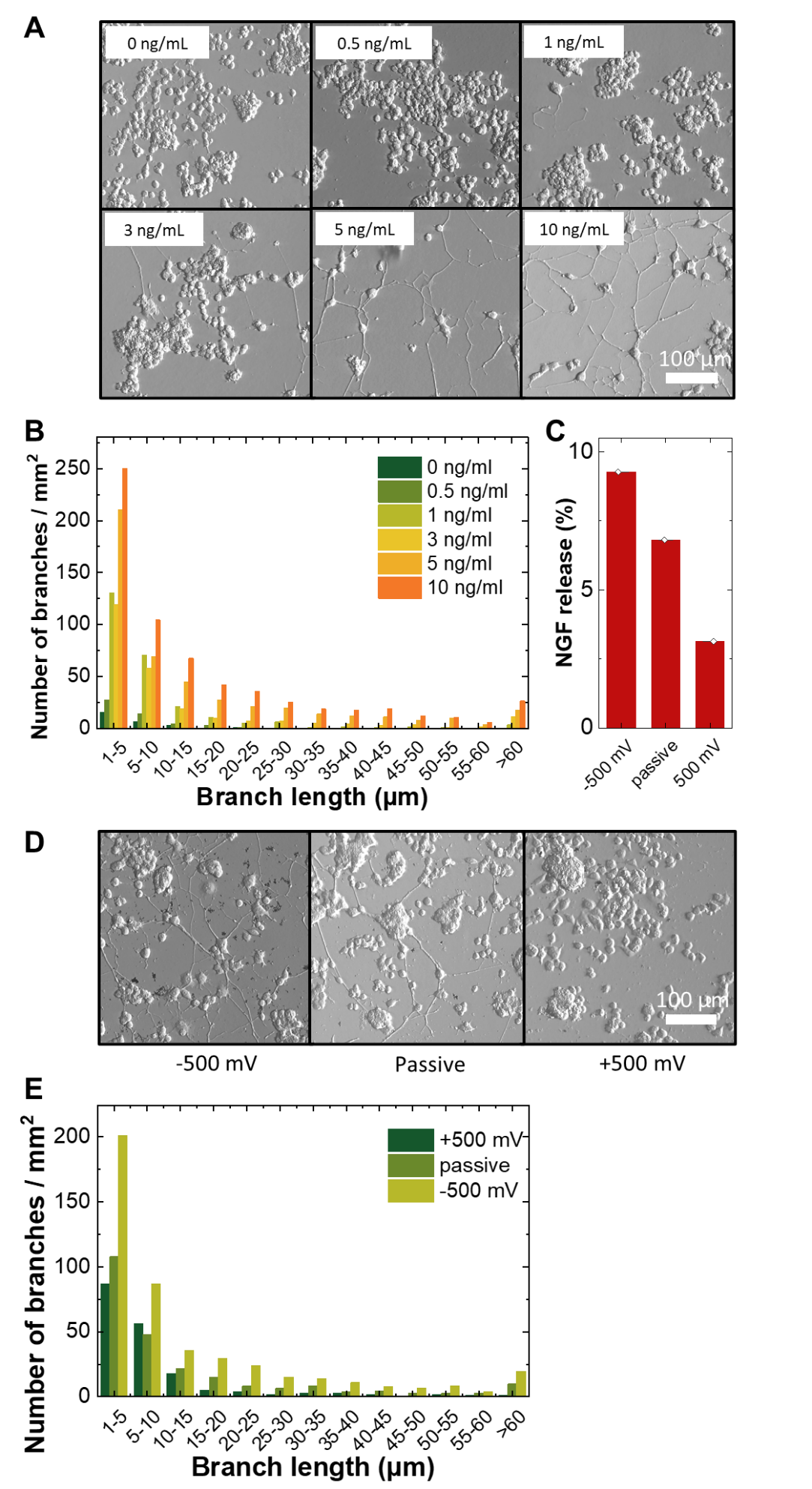


**Figure S17: Determination of NGF loading for PC12 differentiation experiments.**

**(A)** To determine the NGF dose required to stimulate a response, PC12 cells were exposed to varying concentrations of supplemental NGF. Unlike HUVECs, PC12 cells were not embedded in sGAGh so the relevant measure was the growth factor concentration in the media (ng/mL). Using the quantification of neurite density in **(B)**, we determined 5 ng/mL as the minimum effective (threshold) dose needed to stimulate a response in PC12 in this system. Considering the threshold dose, typical uptake fractions in PEDOT:sGAGh, and the volume of cell media, we loaded PEDOT:sGAGh with 1 µg of NGF (from 2.5 mL of PBS + 0.1% BSA). **(C)** Differential release from PEDOT:sGAGh verified that the concentration of NGF passively released over 3 hours is close to the threshold needed to stimulate PC12 cells. Applying reducing or oxidizing potentials then allows us to establish approximately 3-fold difference in the amount of NGF available to PC12 cells. Bar plot shows n = 1 per condition. **(D)** Representative images (phase contrast) of live PC12 cells (5 days after stimulation) following exposure to electrically modulated release of NGF. Active retention (+500 mV) prevents the release of NGF sufficient for neurite growth. **(E)** Quantification of the resulting number density of neurites grown following active retention, active release and passive release of NGF. In the histogram plots (B, E), neurite lengths are grouped into defined bins, and the number of neurites per group is displayed. Neurite lengths were quantified from three independent images per condition (n = 3).


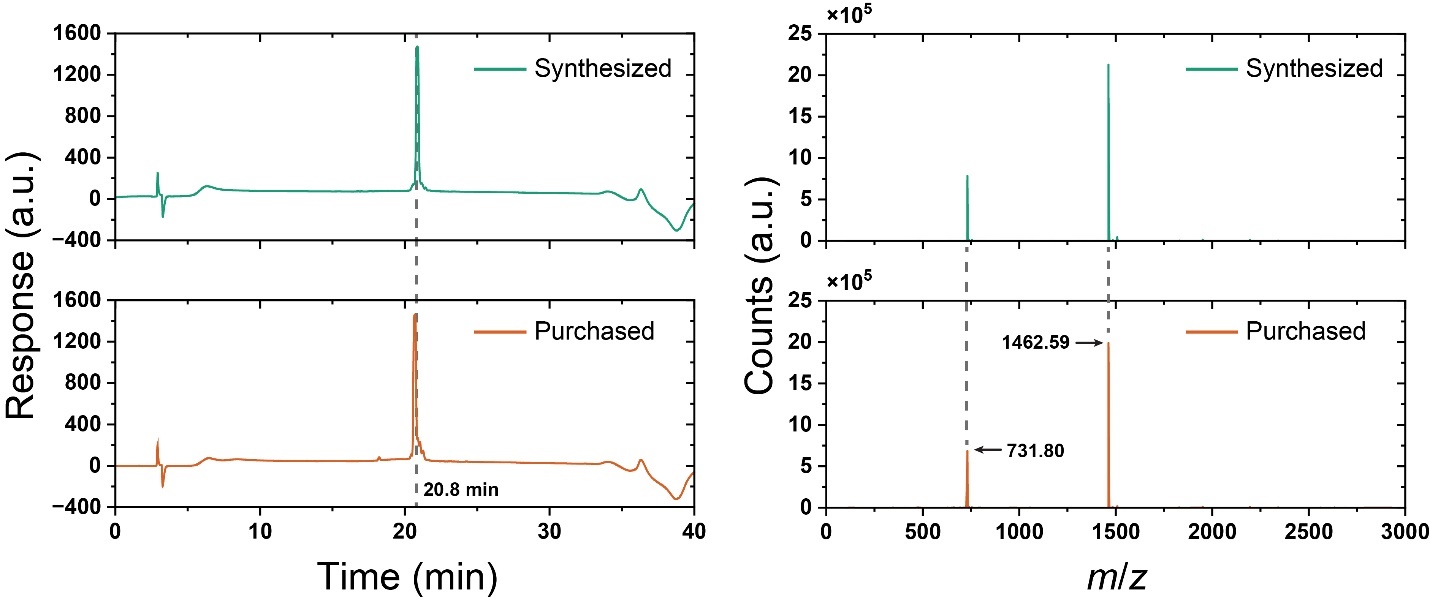


**Figure S18: HPLC-MS of the MMP-cleavable peptide Ac-GCGGPQGIWGQGGCG-NH_2_.**

The MMP-cleavable peptide was either bought or prepared in-house. Both products show identical retention time (HPLC, left) and detected mass (ESI-MS, right). The calculated [*M*+*H*]^+^ and [*M*+2*H*]^2+^ monoisotopic masses for the peptide are 1462.60 and 731.80; the single and doubly charged species are measured as 1462.59 and 731.80 respectively. See the supporting methods for details of the peptide synthesis.

**Supplementary References**

1. Ooi, H. W. *et al.* Thiol-Ene Alginate Hydrogels as Versatile Bioinks for Bioprinting. *Biomacromolecules* 19, 3390–3400 (2018).

2. Kinoshita, M. & Hayashi, T. Accurate and rapid calculation of hydration free energy and its physical implication for biomolecular functions. *Biophys Rev* 12, 469–480 (2020).

3. Sievers-Liebschner, J. *et al.* Unravelling the molecular network structure of biohybrid hydrogels. *Mater Today Bio* 34, (2025).

4. Varongchayakul, N., Huttner, D., Grinstaff, M. W. & Meller, A. Sensing native protein solution structures using a solid-state nanopore: Unraveling the States of VEGF. *Sci Rep* 8, (2018).
